# Development of a forward-oriented therapeutic lentiviral vector for hemoglobin disorders

**DOI:** 10.1038/s41467-019-12456-3

**Published:** 2019-10-02

**Authors:** Naoya Uchida, Matthew M. Hsieh, Lydia Raines, Juan J. Haro-Mora, Selami Demirci, Aylin C. Bonifacino, Allen E. Krouse, Mark E. Metzger, Robert E. Donahue, John F. Tisdale

**Affiliations:** 1 Cellular and Molecular Therapeutics Branch, National Heart Lung and Blood Institute (NHLBI), National Institutes of Health (NIH), Bethesda, Maryland USA; 2 Cellular and Molecular Therapeutics Branch, National Institute of Diabetes and Digestive and Kidney Diseases (NIDDK), NIH, Bethesda, Maryland USA; 3 Translational Stem Cell Biology Branch, NHLBI, NIH, Bethesda, Maryland USA

**Keywords:** Gene therapy, Haematopoietic stem cells, Sickle cell disease

## Abstract

Hematopoietic stem cell (HSC) gene therapy is being evaluated for hemoglobin disorders including sickle cell disease (SCD). Therapeutic globin vectors have demanding requirements including high-efficiency transduction at the HSC level and high-level, erythroid-specific expression with long-term persistence. The requirement of intron 2 for high-level β-globin expression dictates a reverse-oriented globin-expression cassette to prevent its loss from RNA splicing. Current reverse-oriented globin vectors can drive phenotypic correction, but they are limited by low vector titers and low transduction efficiencies. Here we report a clinically relevant forward-oriented β-globin-expressing vector, which has sixfold higher vector titers and four to tenfold higher transduction efficiency for long-term hematopoietic repopulating cells in humanized mice and rhesus macaques. Insertion of Rev response element (RRE) allows intron 2 to be retained, and β-globin production is observed in transplanted macaques and human SCD CD34^+^ cells. These findings bring us closer to a widely applicable gene therapy for hemoglobin disorders.

## Abstract (web summary)

Vectors used in gene therapy for hemoglobin disorders carry globin in a reverse-orientation to prevent the loss of key regulatory elements by RNA splicing, but this limits their efficiency. Here, the authors develop a vector carrying β-globin in a forward orientation and show that it has improved titers and transduction efficiency in humanized mice and nonhuman primates.

## Introduction

Sickle cell disease (SCD) is characterized by a pathogenic hemoglobin (sickle hemoglobin) derived from a point mutation in the β-globin gene, which causes hemoglobin polymerization, sickling of red blood cells (RBCs), hemolytic anemia, vaso-occlusion, end-organ damage, and early mortality. Hydroxyurea (HU) administration is a Food and Drug Administration (FDA)-approved and widely accepted treatment for SCD, which induces fetal hemoglobin expression and thereby reduces sickling^1,2^. However, responses to HU treatment are variable among patients and lead to mostly modest health improvements. Recently, l-glutamine was approved by the FDA as the second available drug for SCD^3^, although the therapeutic effects appear less robust than those of HU. In addition, both l-glutamine and HU require continued usage to maintain their therapeutic effect.

In contrast, allogeneic hematopoietic stem cell (HSC) transplantation with a matched sibling donor can be curative for both pediatric and adult SCD patients. In this approach, SCD patient HSCs are replaced by a one-time infusion of donor HSCs after conditioning and reconstitution of blood production in transplanted patients generates normal RBCs^4–6^. However, a histocompatible donor is only available to around 10% of patients and graft rejection along with graft-vs.-host disease remain significant barriers^7^. An alternative curative strategy is HSC-targeted gene therapy, in which autologous HSCs from SCD patients are modified by adding a normal or therapeutic β-globin gene (or γ-globin gene) using lentiviral vector gene transfer^8,9^. The therapeutic benefits of viral vector-mediated HSC gene therapy have been reported in several gene therapy trials for immunodeficiencies^8,10–13^; however, gene therapy for SCD and β-thalassemia remains under development. Unlike those used in other diseases, therapeutic vectors for the β-globin diseases have demanding requirements including high-level β-globin expression, tissue specificity among erythroid cells, long-term persistence, and high-level modification at the HSC level. These demanding requirements necessitate the inclusion of complex genetic elements including the locus control region (LCR), β-globin promoter, β-globin (or γ-globin) gene, and β-globin downstream region (including the 3′-untranslated region (3′-UTR)), which is now feasible using human immunodeficiency virus type 1 (HIV-1)-based lentiviral vectors^14–17^. The additional requirement of intron 2 for high-level β-globin expression dictates a reverse-oriented globin-expression cassette to prevent the loss of intron 2 by RNA splicing during retroviral vector preparation^15^. Due to the unique requirement for intron 2, globin-containing vectors are the only therapeutic vectors in clinical development that use this reverse orientation, and this strategy has remained unchallenged for decades despite its negative impacts on efficiency.

Current reverse-oriented globin vectors can drive phenotypic correction in mouse models for both β-thalassemia and SCD^14,16,18–22^. Although initial gene therapy trials for β-thalassemia and SCD are encouraging, they also demonstrate the need to improve upon these results by optimizing target cell collection, processing, and transduction^9,23^. Indeed, lower than anticipated gene transfer levels in preliminary gene therapy trials are likely the result of reduced vector copy number (VCN)^24^, as reverse-oriented globin vectors have lower vector titers and lower transduction efficiency in primary human HSCs, which limits their clinical prospects^25^. We hypothesized that reverse orientation impedes both viral preparation and vector transduction.

In this report we describe a forward-oriented globin-expressing vector that retains intron 2, achieves high vector titers, and results in efficient transduction of human HSCs in vitro and in xenograft mice and rhesus HSCs in vivo.

## Results

### Improved titers and transduction with forward orientation

**Fig. 1:**
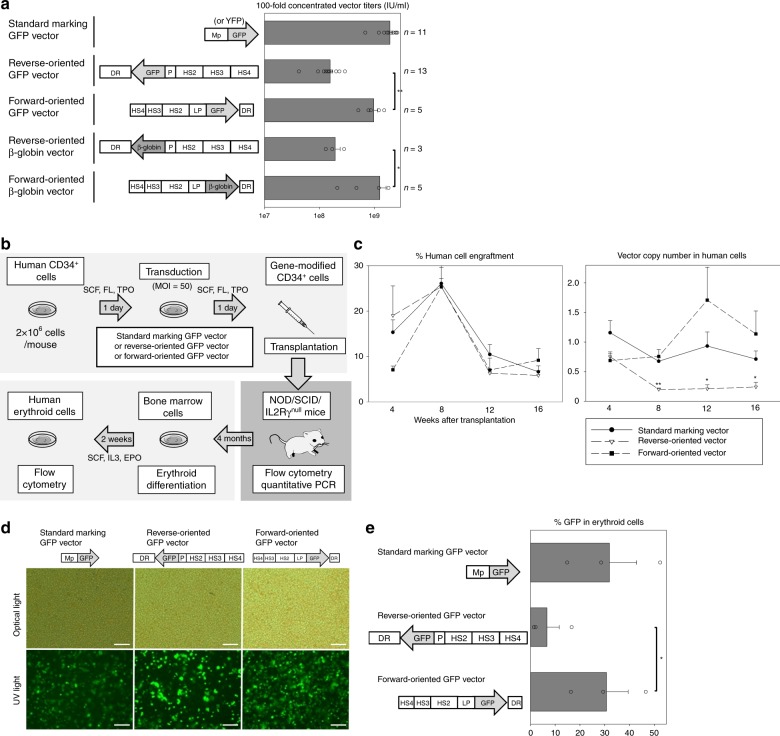
Higher vector titers with 100-fold concentration and more efficient transduction in xenograft mice for the optimized forward-oriented vector for globin expression. **a** We produced the forward-oriented globin-expressing vector with 100-fold concentration, which was optimized by the use of a short β-globin locus control region (LCR) composed of hypersensitive sites 2, 3, and 4 (HS2, HS3, and HS4), a large size of the β-globin promoter (LP), and a β-globin downstream region (DR) lacking the polyA signal. Vector titers of the forward-oriented vectors were evaluated, as compared with the optimized vector in the reverse orientation and a standard GFP-marking vector (*n* = 3–13). **p < 0.01, *p < 0.05 evaluated by *t*-test. **b** We transduced human CD34^+^ cells with reverse-oriented vector, forward-oriented vector, and standard marking vector with a GFP marker gene, and the transduced cells were transplanted into NOD/SCID/IL2Rγ^null^ mice (*n* = 3–5). Four months after transplantation, xenograft bone marrow cells were collected and cultured for human erythroid cells with human specific cytokines. **c** Human cell engraftment and average vector copy numbers (VCNs) in peripheral blood cells were evaluated for 4 months post transplant. **p < 0.01 and *p < 0.05 evaluated by Dunnett’s test, compared with the forward-oriented vector. **d**, **e** After human erythroid cell differentiation from xenograft bone marrow cells (*n* = 3), we evaluated GFP expression using fluorescence microscopy (**d**) as well as GFP-positive percentages in erythroid cells by flow cytometry (**e**). Scale bars represent 100 μm. *p < 0.05 evaluated by *t*-test between forward-oriented and reverse-oriented vectors. EPO: erythropoietin, FL: fms-related tyrosine kinase 3 ligand, GFP: enhanced green fluorescent protein, IL3: interleukin 3, MOI: multiplicity of infection, Mp: murine stem cell virus promoter, P: β-globin promoter, PCR: polymerase chain reaction, SCF: stem cell factor, TPO: thrombopoietin, YFP: enhanced yellow fluorescent protein

Current globin-expressing vectors are all reverse-oriented to prevent the loss of intron 2 by RNA splicing during viral preparation, as intron 2 is required for high-level β-globin expression^15^. We hypothesized that reverse orientation impedes both viral preparation and vector transduction, as despite extensive modifications (described in Supplementary Note 1) through deletion of cryptic polyadenylation (polyA) signals to optimize a conventional reverse-oriented globin vector (yielding a 10-fold increase of vector titers), ~10-fold lower titers and less efficient transduction in human CD34^+^ cells were still observed as compared with a standard forward-oriented gene marking vector (Supplementary Note 1 and Supplementary Fig. 1). We thus designed forward-oriented globin-expressing vectors, in which a globin-expression cassette was inserted in the same orientation as the HIV-1 vector backbone (Supplementary Fig. 2A). Using enhanced green fluorescent protein (GFP) in place of the globin gene, all components in the globin-expression cassette were further optimized for the forward orientation by including a shortened LCR composed of hypersensitive sites 2, 3, and 4 (short HS234), a large-sized β-globin promoter (LP), and a small-sized β-globin downstream region (small DR) including 3′-UTR and lacking both polyA signal and β-globin 3′-enhancer (Supplementary Note 2 and Supplementary Figs. 2, 3, and 4). The 100-fold concentrated vector titers of the forward-oriented vectors (1.0 ± 0.2 × 10^9^ IU/mL) were 6-fold higher than the optimized vector in the reverse orientation (1.6 ± 0.2 × 10^8^ IU/mL, *p* < 0.01) and slightly lower (<2-fold) than that of a standard GFP-marking vector (1.9 ± 0.2 × 10^9^ IU/mL) (Fig. 1a). The forward-oriented vector (including short HS234, LP, and small DR) demonstrated a threefold higher transduction efficiency (%GFP) in human erythroid cells derived from transduced CD34^+^ cells in in vitro culture (51 ± 1%, *p* < 0.01) as compared with the reverse-oriented vector (19 ± 1%) (Supplementary Note 2 and Supplementary Fig. 4B). Evaluation by VCNs resulted in similar trends among vectors (Supplementary Notes 1 and 2, and Supplementary Figs. 1, 2, and 4). These data demonstrate that the optimized forward-oriented vectors for globin expression have higher vector titers and more efficient transduction in CD34^+^ cells for erythroid differentiation than the optimized reverse-oriented vector. We chose the short LCR HS234 LP vector due to the higher transduction efficiency among CD34^+^ cells, and we reasoned that it would provide the highest expression in vivo.

### Improved transduction of human CD34^+^ cells in xenograft mice

To evaluate transduction efficiency in human hematopoietic repopulating cells, human CD34^+^ cells were transduced with a reverse-oriented vector, forward-oriented vector (including short HS234, LP, and small DR), and standard marking vector with a GFP reporter gene (%GFP in vitro after erythroid differentiation 31%, 51%, and 42%, respectively) and the transduced cells were transplanted into immunodeficient NOD/SCID/IL2Rγ^null^ mice (Fig. 1b). We utilized a previously optimized CD34^+^ cell culture condition (1 day pre-stimulation and 1 day transduction), which we developed to allow for efficient lentiviral transduction and high-level engraftment of human CD34^+^ cells^26^. We obtained similar human cell engraftment (human CD45-positive) among all groups (followed for 4 months post transplant) and the forward-oriented vector demonstrated higher VCNs (0.7–1.7) in peripheral blood cells than the reverse-oriented vector (0.2–0.8, *p* < 0.05 at 8, 12, and 16 weeks) and similar VCNs to the standard marking vector (0.7–1.2) (Fig. 1c and Supplementary Fig. 5A). The xenograft bone marrow cells were collected 4 months post transplant and were cultured for human erythroid cells using human-specific cytokines (Fig. 1b). Two weeks after human erythroid cell differentiation, the forward-oriented vector demonstrated fourfold higher GFP-positive percentages (31 ± 9%, *p* < 0.05) as compared with the reverse-oriented vector (7 ± 5%), which was similar to the standard marking vector (32 ± 11%) (Fig. 1d, e and Supplementary Fig. 5A), demonstrating for the first time that this forward-oriented vector performs at the level of our standard constitutively active marking vector.

### Long-term gene marking in transplanted rhesus macaques

**Fig. 2:**
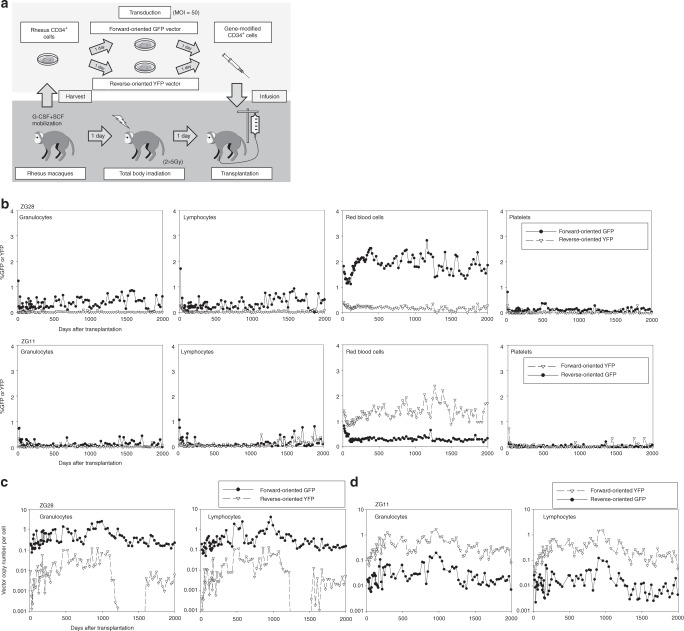
Higher gene marking levels in long-term hematopoietic repopulating cells in rhesus macaques with the forward-oriented vector for globin expression. **a** To evaluate transduction efficiency for long-term hematopoietic repopulating cells, we transduced rhesus CD34^+^ cells with the optimized reverse-oriented vector and forward-oriented vector including a GFP or YFP gene (instead of a globin gene) in a competitive repopulation assay following 10 Gy total body irradiation. **b**–**d** In two animals (ZG28 and ZG11), GFP and YFP gene-marking levels were evaluated by both GFP- or YFP-positive percentages (**b**) and VCNs (**c**, **d**) in peripheral blood cells with the forward-oriented vector up to 5 years, as compared with the reverse-oriented vector. G-CSF: granulocyte colony-stimulating factor

To evaluate transduction efficiency for long-term hematopoietic repopulating cells, we first compared our optimized forward- and reverse-oriented vectors using erythroid elements to drive reporter genes in place of a globin gene. A chimeric HIV-1 vector system was used to efficiently transduce both human and rhesus CD34^+^ cells^27,28^. We transduced rhesus CD34^+^ cells with the optimized reverse-oriented vector and forward-oriented vector including a GFP or enhanced yellow fluorescent protein (YFP) gene at a multiplicity of infection (MOI) 50 in a competitive repopulation assay following 10 Gy total body irradiation (Fig. 2a and Supplementary Fig. 5B). In the first animal (ZG28), half of the CD34^+^ cells were transduced with the forward-oriented vector encoding GFP, whereas the other half were transduced with the reverse-oriented vector encoding YFP. In the second animal (ZG11), the GFP marker was switched to YFP in the forward-oriented vector and vice versa in the reverse-oriented vector as an additional control. Efficient transduction was observed in transduced rhesus CD34^+^ cells before transplantation (%GFP or %YFP 63.4–64.0% and VCNs 2.3–7.1 in the forward-oriented vector, and %GFP or %YFP 41.2–42.9% and VCNs 2.2–10.9 in the reverse-oriented vector) (Supplementary Table 1). After transplantation of the transduced CD34^+^ cells, GFP and YFP signals from both vectors were detected in these two animals exclusively among RBCs by flow cytometry, demonstrating tissue specificity (Fig. 2b). Gene marking levels were evaluated by both GFP- or YFP-positive percentages (Fig. 2b) and VCNs (Fig. 2c, d) in peripheral blood cells. Higher (4- to 10-fold) %GFP or %YFP (ZG28 1.9% vs. 0.2%, *p* < 0.01, and ZG11 1.3% vs. 0.3%, *p* < 0.01, respectively) and 12- to 32-fold higher VCNs (ZG28 0.43–0.52 vs. 0.016–0.017, *p* < 0.01, and ZG11 0.29–0.41 vs. 0.02–0.03, *p* < 0.01, respectively) up to 5 years were demonstrated with the forward-oriented vector, as compared with the reverse-oriented vector. In addition, we confirmed higher levels of GFP or YFP RNA expression from the forward-oriented vector in differentiated erythroid cells from both animals at extended follow-up (Supplementary Fig. 6).

**Fig. 3:**
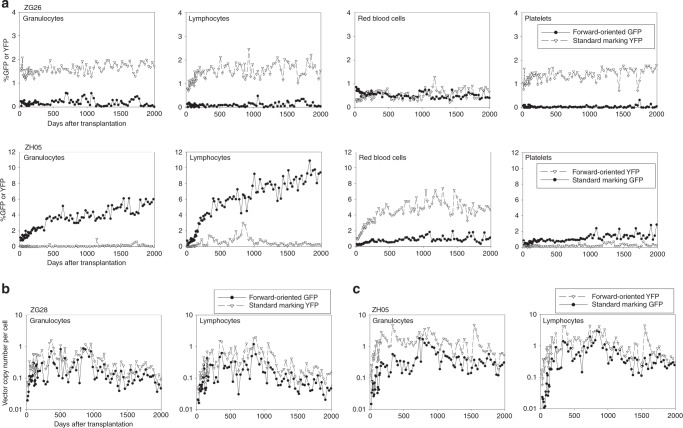
The forward-oriented vector achieves long-term marking levels in long-term hematopoietic repopulating cells in rhesus macaques similar to a standard gene marking vector. **a**–**c** We compared the forward-oriented vector with a standard gene marking vector including a GFP or YFP gene in the rhesus competitive repopulation assay. In two animals (ZG26 and ZH05), GFP and YFP gene-marking levels with the forward-oriented vector were evaluated by GFP- or YFP-positive percentages (**a**) and VCNs (**b**, **c**) for 5 years, as compared with a standard GFP or YFP-marking vector

As variability is common among outbred animals, we sought to determine how this forward-oriented vector compared with our standard GFP-marking vector, for which we often see clinically relevant gene marking in vivo long term using the competitive repopulation model. We thus compared the forward-oriented erythroid-specific vector with our standard gene-marking vector including a GFP or YFP gene driven by a constitutive promoter in a rhesus competitive repopulation assay. The forward-oriented vector and standard marking vector encoded GFP and YFP genes, respectively, in the third animal (ZG26), and YFP and GFP genes in the fourth animal (ZH05), and efficient transduction was observed in rhesus CD34^+^ cells in vitro (%GFP or %YFP 74.7–79.6% and VCNs 2.2–4.1 in the forward-oriented vector, and %GFP or %YFP 52.5–63.6% and VCNs 0.8–3.4 in the standard marking vector) (Supplementary Table 1). After transplantation of the transduced CD34^+^ cells, standard marking vectors demonstrated multi-lineage GFP or YFP expression in both ZG26 (granulocytes 1.6%, lymphocytes 1.5%, RBCs 0.5%, and platelets 1.4%) and ZH05 (granulocytes 3.3%, lymphocytes 5.3%, RBCs 0.8%, and platelets 1.1%), whereas erythroid-specific GFP or YFP expression was observed again from the LCR-driven forward-oriented vectors by flow cytometry (Fig. 3a). Gene-marking levels with the forward-oriented vector were comparable to standard GFP- or YFP-marking vectors for 5 years, demonstrating a one- to fourfold difference in GFP- or YFP-positive percentages (ZG26 0.542% vs. 0.541%, ns, and ZH05 4.1% vs. 0.8%, *p* < 0.01, respectively) (Fig. 3a) and a two- to fourfold difference in VCNs (ZG26 0.15–0.18 vs. 0.37–0.40, *p* < 0.01, and ZH05 1.16–1.31 vs. 0.34–0.47, *p* < 0.01, respectively) (Fig. 3b, c). In addition, similar in vitro GFP or YFP RNA expression was observed in the forward-oriented and standard marking vectors in differentiated erythroid cells from both animals (Supplementary Fig. 6). These data demonstrate that the forward-oriented vector for globin expression achieves efficient transduction comparable to our standard marking vectors for long-term hematopoietic repopulating cells with erythroid-specific transgene expression.

### Positive selection of intron 2-containing β-globin vector

**Fig. 4:**
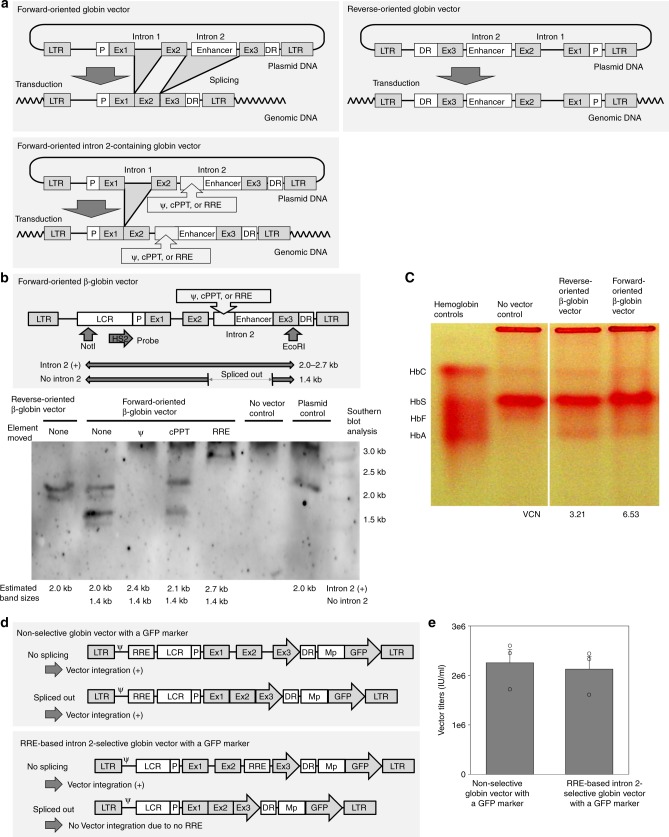
Insertion of the Rev response element (RRE) allows for positive selection of intron 2-containing β-globin vector. **a** To positively select intron 2-containing β-globin vectors, essential viral components (packaging signal (0.4 kb), central polypurine tract (cPPT) (0.1 kb), or RRE (0.8 kb)) were deleted in the backbone of the forward-oriented vector and the deleted viral components were inserted into intron 2 of the β-globin gene. **b** We evaluated integrating vector sizes by Southern blot analysis, to determine intron 2-positive (2.0–2.9 kb) and no intron 2 (1.4 kb) percentages for the forward-oriented β-globin vectors with or without intron 2 selection. **c** We confirmed β-globin expression from the forward-oriented vector by adult hemoglobin (HbA) production in human erythroid cells derived from transduced peripheral blood mononuclear cells in sickle cell disease (SCD). **d** We designed both the intron 2-selective vector and non-selective vector using a forward-oriented globin vector construct with an additional GFP-expression cassette. The intron 2-selective vector would be integrated and express GFP only when both intron 2 and RRE were intact (not spliced out). The non-selective vector should be integrated with or without intron 2. **e** We evaluated vector titers based on GFP positivity for the intron 2-selective vector (only intron 2-positive) and non-selective vector (overall vector production) in the forward-oriented β-globin construct. n.s. evaluated by *t*-test. HbC: hemoglobin C, HbS: sickle hemoglobin, HbF: fetal hemoglobin, LTR: long terminal repeat, ψ: packaging signal. All experiments except Southern blotting and Hb electrophoresis (single run) were performed in triplicate

We then replaced the GFP gene with the β-globin gene containing intron 2 in the optimized forward-oriented vector construct. To prevent the loss of intron 2 by RNA splicing during viral vector preparation in the forward orientation, we devised a strategy to ensure that only intron 2-containing vector would be packaged. To positively select intron 2-containing β-globin vectors, essential viral components (packaging signal, central polypurine tract (cPPT), or Rev response element (RRE)) were deleted in the backbone of the forward-oriented vector and the deleted viral components were inserted into intron 2 of the β-globin gene (Fig. 4a). We transduced HeLa cells with forward-oriented β-globin gene vectors with or without these selectable components (packaging signal (0.4 kb), cPPT (0.1 kb), or RRE (0.8 kb)) in intron 2 and evaluated the intron 2-containing vector and no intron 2 vector in transduced cells by Southern blot analysis (Fig. 4b and Supplementary Fig. 7A). We observed that around half of the forward-oriented vectors lost intron 2 during vector preparation when no elements were included in intron 2 (43% of intron 2-containing vector), whereas insertion of the RRE resulted in a positive selection (~100%) of intron 2-containing β-globin vectors. The cPPT insertion demonstrated little effect on selection of intron 2-containing vectors (59%), whereas lentiviral vectors created by insertion of the packaging signal into intron 2 did not integrate, suggesting the packaging signal is disrupted by changing its position in the vector construct. We confirmed β-globin expression from the forward-oriented RRE-based vector by adult hemoglobin production at the protein level among human erythroid cells derived from transduced peripheral blood mononuclear cells (PBMCs) in SCD (Fig. 4c and Supplementary Fig. 7B).

For further analysis of intron 2 selection, we designed both an intron 2-selective vector and a non-selective vector using a forward-oriented globin vector construct with an additional GFP-expression cassette (Fig. 4d). The intron 2-selective vector would be integrated and express GFP only when both intron 2 and RRE were intact (not spliced out), whereas the non-selective vector should be integrated to express GFP with or without the intron 2. We evaluated GFP-based vector titers between the intron 2-selective vector (only intron 2-positive) and the non-selective vector (overall vector production) in the forward-oriented β-globin construct (Fig. 4e). We observed similar titers between the intron 2-selective vector and the non-selective vector in the forward-oriented β-globin vector construct, suggesting that RRE insertion allows efficient intron 2 selection for high-titer vector preparation with the forward-oriented β-globin gene.

### Human β-globin production in transplanted rhesus macaques

**Fig. 5:**
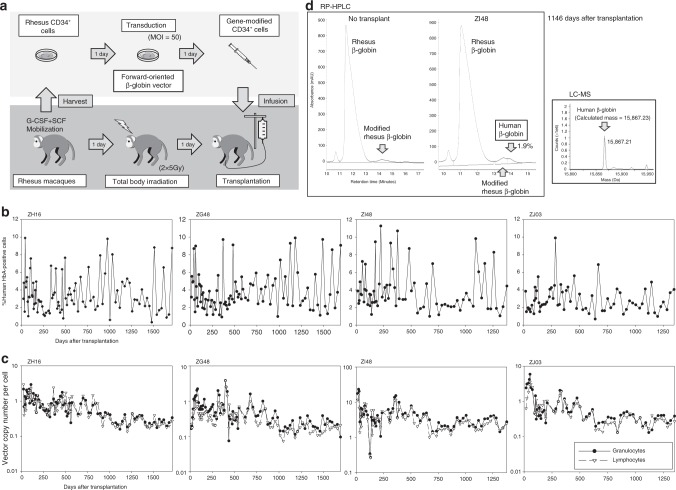
Detectable human β-globin production at the protein level with the forward-oriented β-globin vector in rhesus transplantation. **a** We transduced rhesus CD34^+^ cells with the optimized forward-oriented β-globin vector (with RRE-based intron 2 selection) at MOI 50, and these cells were transplanted into autologous animals (ZH16, ZG48, ZI48, and ZJ03) following 10 Gy total body irradiation. **b** In all animals, human β-globin expression was analyzed in RBCs for 4 years post transplant, evaluated by a human HbA antibody in flow cytometry. **c** Gene marking levels were evaluated in both granulocytes and lymphocytes up to 4 years. **d** Three years post transplant in ZI48, human β-globin production in RBCs was evaluated at the protein level by reversed-phase high-performance liquid chromatography (RP-HPLC) and by liquid chromatography-mass spectrometry (LC-MS). All globin protein analysis experiments were performed in a single run

When the GFP gene was switched to the β-globin gene with RRE selection, the 100-fold concentrated vector titers for forward-oriented β-globin vectors (1.3 ± 0.4 × 10^9^ IU/mL) were still sixfold higher than reverse-oriented β-globin vectors (1.9 ± 0.4 × 10^8^ IU/mL, *p* < 0.05) (Fig. 1a) and nearly 100-fold higher when compared with our original reverse-oriented β-globin vectors. We transduced rhesus CD34^+^ cells with the optimized forward-oriented β-globin vector (with RRE-based intron 2 selection) at MOI 50 (VCNs 1.3–8.6) (Supplementary Table 1) and these cells were transplanted into autologous animals (ZH16, ZG48, ZI48, and ZJ03) following 10 Gy total body irradiation (Fig. 5a). In all animals, human β-globin expression was detected in RBCs for 4 years post transplant (ZH16 3.5%, ZG48 3.9%, ZI48 3.9%, and ZJ03 2.9%), as evaluated by detection using a human adult hemoglobin antibody in flow cytometry (Fig. 5b and Supplementary Fig. 5B). We observed stable gene marking in both granulocytes and lymphocytes up to 4 years (VCNs: ZH16 0.76 and 0.75, ZG48 0.62 and 0.43, ZI48 4.75 and 3.76, and ZJ03 0.84 and 0.70, respectively) (Fig. 5c). Human β-globin production was confirmed by reversed-phase high-performance liquid chromatography (RP-HPLC) (Fig. 5d), as well as liquid chromatography-mass spectrometry (LC-MS) (Fig. 5d) 3 years post transplant in ZI48. In addition, human β-globin expression was observed at the RNA level in bone morrow cells in ZI48 (Supplementary Fig. 8). These data demonstrate that the forward-oriented β-globin vector with intron 2 selection allows for efficient transduction among long-term hematopoietic repopulating cells at levels comparable to standard marking vectors lacking these complex control elements, as well as detectable human β-globin production at the protein level in a rhesus gene therapy model.

### βT87Q-globin production from transduced SCD CD34^+^ cells

We further improved the forward-oriented vector by including β-globin 3′-enhancer and additional hypersensitivity sites 1 and 5 (HS1–5) to increase transgene expression (Supplementary Fig. 9) as compared with the formerly optimized forward-oriented vector including HS2, HS3, and HS4 (HS234) (described in the Supplementary Note 3). The HS1–5 forward-oriented vector has higher vector titers (*p* < 0.05), higher transduction efficiency (%GFP, *p* < 0.01 and VCNs, *p* < 0.05), and higher transgene expression levels (GFP intensity, *p* < 0.05), as compared with the reverse-oriented vector (Supplementary Note 3 and Supplementary Fig. 9), demonstrating that the HS1–5 forward-oriented vector allows for high vector titer, high-efficiency transduction in human CD34^+^ cells, and high-level transgene expression in erythroid cells.

**Fig. 6:**
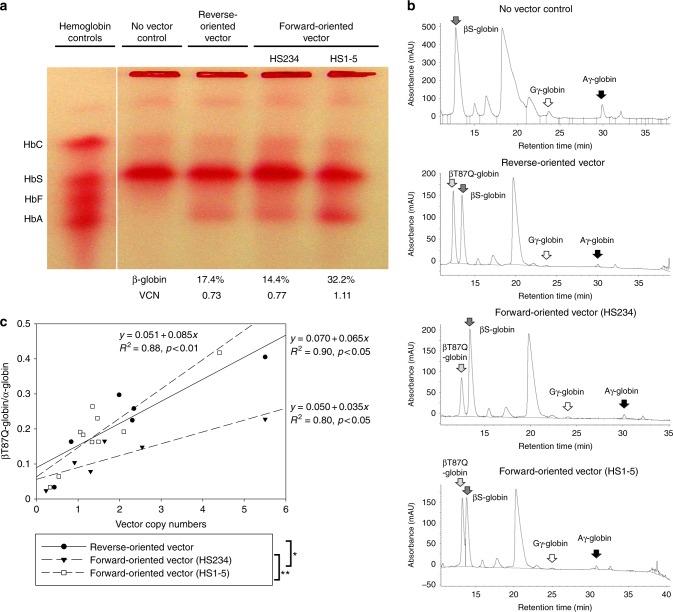
Robust β-globin production at the protein level with the forward-oriented β-globin vector in erythroid cells differentiated from plerixafor-mobilized CD34^+^ cells in SCD. **a** SCD CD34^+^ cells were transduced with the optimized forward-oriented β-globin (or βT87Q-globin including an anti-sickling mutation T87Q) vectors at MOI 50 and the transduced cells were differentiated into erythroid cells for 2 weeks. β-globin production was evaluated by hemoglobin electrophoresis (HbA bands) and/or RP-HPLC (%), and VCNs were evaluated by quantitative PCR. **b** Globin protein peaks were detected by RP-HPLC. **c** Globin-expression levels per vector were evaluated by regression lines between VCNs and βT87Q-globin amounts at the protein level. The βT87Q-globin amounts were calculated by the area under the curve in RP-HPLC, compared with α-globin. **p < 0.01 and *p < 0.05 evaluated by Tukey’s HSD test among regression lines. All globin protein analysis experiments were performed in a single run

To analyze vector-derived globin expression levels in erythroid cells, we transduced CD34^+^ cells collected after plerixafor mobilization in patients with SCD with the optimized forward-oriented vectors encoding β-globin as well as βT87Q-globin (including an anti-sickling mutation T87Q) at MOI 50^29^. We transduced SCD CD34^+^ cells 2 days after initiating erythroid differentiation and 2 weeks later, we evaluated hemoglobin production by hemoglobin electrophoresis and/or RP-HPLC. In the no transduction control, we exclusively observed a sickle hemoglobin band by hemoglobin electrophoresis, whereas transduction with the reverse-oriented and the forward-oriented globin vectors (encoding either normal β-globin or βT87Q-globin) resulted in an additional adult hemoglobin band (Fig. 6a and Supplementary Fig. 7C). An additional peak for βT87Q-globin was detected for both the reverse-oriented and the forward-oriented vectors by RP-HPLC (Fig. 6b). Quantification of vector-derived βT87Q-globin production demonstrated up to 48% of the total β-globin series with the HS1–5 forward-oriented vector (Fig. 6b). Globin-expression level per vector was evaluated by the ratios between βT87Q-globin protein amounts and VCNs in serial human CD34^+^ cell transduction followed by erythroid differentiation. The HS1–5 forward-oriented vector improved βT87Q-globin production per vector as compared with the HS234 forward-oriented vector (*p* < 0.01), and similar βT87Q-globin production per VCN was observed between the HS1–5 forward-oriented vector and reverse-oriented vector (Fig. 6c). These data demonstrated that the forward-oriented β-globin vector with intron 2 selection allows for efficient transduction as well as robust globin expression in human erythroid cells derived from SCD subjects after plerixafor mobilization.

## Discussion

We have developed a forward-oriented β-globin-expressing vector resulting in sixfold higher vector titers and four- to tenfold higher VCN among long-term hematopoietic repopulating cells and their progeny when compared with our optimized, reverse-oriented β-globin-expressing vector. We reasoned that the relatively low titers and transduction efficiencies of lentiviral vectors driving expression of β-globin are related to the necessity of a reverse orientation to prevent the loss of intron 2, which is important for achieving high-level β-globin expression. This reverse orientation is unique among all other therapeutic lentiviral vectors. Although titers were increased remarkably by preparing these vectors in the forward orientation, we found that about half of the vectors lacked intron 2 when prepared in the forward orientation. To circumvent this loss, we inserted various elements required for vector production into intron 2, with insertion of the RRE, which is necessary for packaging, allowing for successful selection of intron 2-containing forward-oriented vector. Prolonged follow-up over 4 years post transplant in rhesus macaques demonstrates superior transduction of true long-term repopulating HSCs with this vector system. The nearly 100-fold improvement in vector titers that we obtained compared with our original vectors reduced both time and cost of large-scale vector production, which renders this vector system highly applicable for clinical use. It should also be noted that the improved transduction efficiency of our forward-oriented vector compared with reverse-oriented vectors is based on transductions performed at identical MOI, meaning that not only is production cost and effort reduced, the potency of the vector is increased as well. As VCN has recently been demonstrated as a limiting factor in lentiviral vector-mediated gene transfer and transplantation for SCD patients, the improved efficiency of HSC transduction observed using this construct should improve the therapeutic potential of gene therapy for SCD and other hemoglobin disorders. The RRE insertion allows us to produce intron 2-containing forward-oriented β-globin vector, permitting long-term human β-globin expression detectable at the protein level in rhesus macaques.

We initially sought to increase vector titers by simply using a forward orientation instead of the traditional reverse orientation, because we hypothesized that increased vector titers are required for efficient transduction of CD34^+^ cells. Low-titer lentiviral vectors pseudotyped with a vesicular stomatitis virus G protein (VSVG) envelope contain greater amounts of non-functional vector particles than high-titer vectors^25^. Non-functional particles in low-titer vectors likely interfere with the internalization of functional particles by blocking VSVG receptors on HSCs, resulting in less efficient transduction. Indeed, a low-titer reverse-oriented vector could not efficiently transduce human CD34^+^ cells even with high MOIs (Supplementary Fig. 1), probably due to saturation of VSVG receptors on CD34^+^ cells by non-functional particles. On the other hand, high-titer forward-oriented vectors efficiently transduced human CD34^+^ cells with more efficient transduction at increasing MOIs (Supplementary Fig. 2). These data suggest that high titers (and high vector concentration) are important for efficient transduction in human CD34^+^ cells.

We then evaluated gene marking levels in transplanted animals after long-term multi-lineage reconstitution, because long-term models of HSC transplantation are the most reliable for evaluating HSC transduction. However, rhesus HSCs respond differently than human HSCs when transduced with HIV-1-based lentiviral vectors, because HIV-1 infection is restricted by innate immunity factors^30,31^. Therefore, we previously developed a humanized xenograft mouse model with lentiviral transduction, which allows for human hematopoietic reconstitution with lentiviral gene marking in transplanted mice^26^. In the xenograft mouse model, we demonstrated that cytokine stimulation results in more efficient lentiviral transduction but lower engraftment of human CD34^+^ cells; thus, we optimized CD34^+^ cell culture conditions (1 day pre-stimulation and 1 day transduction) to accomplish both efficient lentiviral transduction and high-level engraftment^26^. In addition, we circumvented the restriction to HIV-1-based lentiviral vectors in the rhesus HSC gene therapy model by developing a chimeric HIV-1-based lentiviral vector including the simian immunodeficiency virus capsid, which allows for efficient transduction in both human and rhesus CD34^+^ cells^27,28^. In this study, these animal models were used to evaluate levels of transduction with forward-oriented globin vectors in human and rhesus cells following hematopoietic reconstitution in transplanted animals (Figs. 2, 3, and 5). Our data demonstrate more efficient transduction of both human and rhesus HSCs with the forward-oriented globin vector, as compared with the reverse-oriented globin vector. Importantly, these levels are equivalent to our standard marking vector lacking the complex globin regulatory elements that have historically limited both vector titer and transduction efficiency. The more efficient transduction and higher VCN consistently observed with the forward-oriented globin vector should improve the therapeutic potential of lentiviral gene therapy strategies already in development for hemoglobin disorders.

We demonstrated substantially higher gene-marking levels with the forward-oriented globin-expressing vectors for both differentiated erythroid cells in vitro and long-term hematopoietic repopulating cells in rhesus macaques. We also demonstrated similar transgene expression levels in forward-oriented globin vectors evaluated by both GFP intensity and adult hemoglobin production in differentiated erythroid cells in vitro, as compared with the reverse-oriented globin vector (Figs. 4 and 6, and Supplementary Figs. 2, 4, and 9). These data suggest sufficient amounts of globin production for phenotypic correction could be achieved using the forward-oriented globin vector, as reverse-oriented globin vectors have allowed for phenotypic correction in mouse models and preliminary clinical trials for hemoglobin disorders^8,9,14,16,18–23^. Around 20% gene marking in HSCs and 20% β-globin production in RBCs are thought to be required for SCD gene therapy, based on modeling of donor chimerism after allogeneic HSC transplantation in SCD at our center^32^. In a current SCD gene therapy trial (with a reverse-oriented vector), VCNs of 1–2 in PBMCs appear sufficient to direct therapeutic effects for SCD, whereas lower VCNs do not^23,24^. We demonstrated higher gene-marking levels with our forward-oriented vectors than a reverse-oriented vector, achieving VCNs of 0.4–4.8 with the forward-oriented β-globin vector in a total of 4 rhesus macaques (ZH16 0.75–0.76, ZG48 0.43–0.62, ZI48 3.76–4.75, and ZJ03 0.70–0.84). However, we anticipate the levels of human β-globin production observed in rhesus RBCs to be lower than those in a SCD gene therapy setting, as the extended lifespan of corrected RBCs would allow for their selection in SCD patients, whereas this survival advantage should not be reflected in the rhesus model^33^. In experiments using plerixafor-mobilized SCD CD34^+^ cells, similar amounts of β-globin production were observed using the forward-oriented and reverse-oriented vector, achieving ~50% of β-globin production (Fig. 6b). These data suggest that the forward-oriented β-globin vector can improve transduction efficiency of HSCs and direct sufficient β-globin production to allow successful SCD gene therapy.

Currently, reverse-oriented globin vectors are used in all gene therapy trials for hemoglobin disorders, including β-thalassemia and SCD^9,23^. In preliminary gene therapy trials, partial therapeutic effects (reduction of RBC transfusion) were observed for β-thalassemia patients^9^. However, once higher levels of gene transfer were obtained (attributed to improved transduction efficiency among individuals), improvements were seen in β-thalassemia and in a single SCD subject, demonstrating the importance of efficient transduction for improving prospects of gene therapy in hemoglobin disorders^9,23,24,34,35^. Improving transduction efficiency of HSCs appears even more critical in gene therapy for SCD as compared with β-thalassemia, as marking levels have thus far been lower in SCD gene therapy trials. Furthermore, β-globin protein levels achieved by reverse-oriented vectors to date may be insufficient to overcome the pathogenic effects of sickle globin, which are not mitigated by the gene addition approach. The more efficient transduction of CD34^+^ cells in vitro and in vivo achieved with our forward-oriented globin vector (Figs. 1, 2, and 3, and Supplementary Figs. 2 and 4) should advance gene therapy for hemoglobin disorders by allowing us to achieve transduction levels required for broad success in SCD.

In parallel, we (and others) are investigating other promising strategies to increase transduction efficiency in gene therapy for SCD. We previously optimized conditions for CD34^+^ cell transduction with lentiviral vectors using a xenograft mouse model and demonstrated efficient long-term gene marking in a rhesus gene therapy model^26–28^. Current conditions are highly optimized including the time course, cytokine stimulation, and culture media; however, further improvements in transduction efficiency for CD34^+^ cells have been developed in the interim. It is important, therefore, to compare the results with our control vectors, to appreciate the level of improvement this vector system allows. Additional measures may also be required to advance gene therapy for SCD. We recently determined that a higher fraction of erythroid progenitors are contained within the CD34^+^ cell fraction in the marrow of SCD patients, suggesting improved collection and purification methods might be required for SCD gene therapy^36^. Optimized CD34^+^ cell collection and transduction will be combined with our efficient forward-oriented globin vector system as a next step.

Intron 2 has historically been included in all β-globin vectors since Miller’s pioneering work in the 1980s, demonstrating its importance for expression and consequently the requirement for reverse orientation to prevent its loss by RNA splicing. Thus, we devised a strategy to ensure that this element was not removed during viral preparation in the forward orientation. After testing insertion of several critical viral vector elements, we determined that insertion of the RRE into intron 2 allows for positive selection of intron 2-containing forward-oriented globin vector among transduced cells (Fig. 4). We confirmed human β-globin protein production in the forward-oriented β-globin vector with RRE selection in differentiated erythroid cells from normal volunteers and from patients with SCD by hemoglobin electrophoresis and RP-HPLC, and in a rhesus macaque 3 years post transplant by RP-HPLC and LC-MS (Figs. 4, 5, and 6). These data suggest that the forward-oriented β-globin vector with RRE selection allows for efficient transduction in HSCs, and that RRE insertion into intron 2 does not interfere with β-globin protein production.

In reverse-oriented vectors, inclusion of promoter, coding region, and polyA signal is required for transgene expression. In contrast, the polyA signal should be removed in forward-oriented vectors, as the 3′-long terminal repeat (LTR) is utilized as a polyA signal in forward-oriented vectors. The presence of an internal polyA signal possibly can result in bidirectional termination that may interfere with transcription of the full-length vector RNA genome during lentiviral vector preparation, reducing vector titers^37^. Basically, polyA signals induce cleavage of mRNA and the addition of a polyA tail in only one direction; however, a bidirectional termination is also observed in polyA signals^38–40^. In a reverse-oriented globin vector, polyA function was reported in the opposite direction of the downstream region (including a polyA signal)^41,42^. In addition, cryptic polyA signals can be produced in unexpected regions when switching from forward orientation to reverse orientation in globin vector constructs^42^. Therefore, the β-globin downstream region as well as cryptic polyA signals could reduce vector titers in reverse-oriented vectors. In contrast, a forward-oriented β-globin coding region should be optimal for RNA transcription, as RBCs are capable of high-level hemoglobin production. In addition, insertion of woodchuck hepatitis virus post-transcriptional regulatory element (WPRE) was reported to improve lentiviral vector titers^43^ and the WPRE fragment was utilized for previous optimization in reverse-oriented β-globin vectors (upstream or downstream of 3′-LTR)^44,45^, suggesting that vector RNA stability might be improved by WPRE insertion, which might be a potential reason for the low titers in reverse-oriented vectors.

MOI escalation (from 1 to 50) of the reverse-oriented vector resulted in increased %GFP (from 6 to 11%) and VCNs (from 0.04 to 0.4), but no increase in GFP intensity (~1300) in the GFP-positive fraction (Supplementary Fig. 1C). In low-level transduction (6–11%) with the reverse-oriented vector at MOIs 1–50, most of the transduced cells should have 1 vector genome per cell; therefore, GFP intensity in GFP-positive fraction should not increase by MOI escalation. In contrast, an increase in %GFP (from 13 to 64%), VCNs (from 0.01 to 0.8), and GFP intensity (from 300 to 800) was observed with the standard marking vector, as high-level transduction (64%) at MOI 50 should increase the amounts of vector genome per cell (1 or more) in the transduced cell fraction, resulting in higher GFP intensity in the GFP-positive fraction. In this MOI escalation, similar trends were observed between %GFP and VCNs; both %GFP and VCNs in the standard marking vector increased more sharply at higher MOIs as compared with the reverse-oriented vector. However, MOI escalation (1–50) increased VCNs (10–80-fold) more strongly than %GFP (2–5-fold) in both vectors. %GFP is saturated up to 100%, whereas VCNs can increase >1; thus, VCNs can increase more sharply in MOI escalation than %GFP.

We analyzed the correlation between in vitro VCNs in transduced CD34^+^ cells and in vivo VCNs in granulocytes and lymphocytes, as well as in vitro %GFP (or %YFP) in differentiated erythroid cells and in vivo VCNs in transplanted rhesus macaques. We observed a positive correlation between in vitro %GFP (or %YFP) and in vivo VCNs (*R*^2^ = 0.53, *p* < 0.05 in granulocytes and *R*^2^ = 0.47, *p* = 0.06 in lymphocytes); however, we failed to show a strong correlation between in vitro VCNs and in vivo VCNs (*R*^2^ = 0.03, *p* = 0.60 in granulocytes and *R*^2^ = 0.02, *p* = 0.69 in lymphocytes). We have previously demonstrated a positive correlation between in vitro %GFP and in vivo VCNs, as well as in vitro VCNs and in vivo VCNs in our rhesus transplantation model with lentiviral transduction; however, in vitro VCNs can be overestimated by contaminated vector plasmids that are used for vector preparation, reducing their predictive value^46^. We utilized an integration-specific probe/primers for VCN evaluation, but when large amounts of plasmid or non-integrated vector DNA (pre-integration complex) remain in transduced cells, this method is less reliable for determining in vitro VCNs. Indeed, in an ongoing gene therapy trial for SCD, improving VCN in vitro seems important to enhance the therapeutic potential of lentiviral gene transfer strategies in patients^24^.

For the first time, we demonstrated human β-globin production at the protein level detectable in RP-HPLC 3 years after rhesus transplantation of transduced CD34^+^ cells (Fig. 5d). This additional peak of human β-globin protein was analyzed by mass spectrometry to confirm the specific protein mass (15867.2), which was matched to the calculated mass based on the amino acid sequence (Fig. 5d). We confirmed human β-globin RNA expression in bone marrow cells in the transplanted rhesus macaques (Supplementary Fig. 8). We also observed low positivity for GFP (or YFP) in non-erythroid cells from both reverse-oriented and forward-oriented vectors including HS234 and β-globin promoter. The presence of small amounts of transgene products in leukocytes may be important for education of immune cells to recognize GFP (or YFP) as a self-protein. Detection of human β-globin production should be more difficult in rhesus macaques as compared with patients with SCD, as SCD RBCs have shorter half-life than normal RBCs^32^. Thus, normal or therapeutic β-globin gene addition should allow for positive selection of transduced RBCs with longer half-life in gene therapy patients, but this positive selection should not occur in rhesus macaques. We further improved transgene expression levels by using the HS1–5 forward-oriented vector including additional HS1 and HS5, as well as β-globin 3′-enhancer (Fig. 6 and Supplementary Fig. 9). In addition, we recently demonstrated more efficient transduction methods in human CD34^+^ cells in high-density culture with adjuvants^47^. Improvement of transduction efficiency and transgene expression might allow for higher amounts of human β-globin production at the protein level in transplanted rhesus macaques and, ultimately, in humans.

We performed a large number of rhesus transplantation experiments with gene marking followed for more than 10 years, and stable gene marking was achieved around 6 months after transplantation^27,28,48^. However, slight increases or decreases in gene-marking levels are often observed by %GFP and/or VCNs even beyond 6 months after transplantation. In parallel, we routinely checked complete blood counts to confirm normal hematopoiesis. In this study, we observed mild increases or decreases in gene-marking levels by %GFP (or %YFP) and VCNs, but these fluctuations likely reflect normal hematopoiesis.

In summary, we have developed a clinically relevant, forward-oriented, globin-expressing vector, with marked improvements in both viral titer and transduction efficiency for hematopoietic repopulating cells, as compared with an optimized reverse-oriented vector similar to those being studied in current clinical trials in humans. RRE insertion allowed positive selection of intron 2-containing forward-oriented β-globin vectors and human β-globin production was observed in transplanted rhesus macaques with the forward-oriented β-globin vector transduction. These findings bring us closer to a curative gene therapy approach for hemoglobin disorders.

## Methods

### Lentiviral vector design and preparation

A chimeric HIV-1-based lentiviral vector (χHIV vector) system was used to efficiently transduce both human and rhesus hematopoietic cells, as previously described^27,28,31,49,50^. The erythroid-specific globin expression vectors contain the LCR (HS1 (GenBank sequence U01317; 0.2 kb: 13337–13535), HS2 (0.8 kb: 8049–8864), HS3 (0.7 kb: 4475–5176, 0.9 kb: 4300–5177, or 1.3 kb: 3877–5177), HS4 (0.2 kb: 1025–1224, 0.4 kb: 957–1390, or 1.1 kb: 328–1390), and/or HS5 (NG_052895; 0.4 kb: 21762–21611 and 20321–20090)), β-globin promoter (0.2 or 0.7 kb), β-globin gene with introns (0.4 kb deletion within the intron 2 (+579 to  +614)) or GFP (or YFP) gene, β-globin downstream region including 3′-UTR with or without a polyA signal (0.7 kb or 0.1 kb, respectively), and/or β-globin 3′-enhancer (U01317: 0.3 kb insertion in the forward-oriented vector: 64297–64561, 0.3 kb deletion in the reverse-oriented vector: 64290–64620)^17,25^. To positively select intron 2-containing β-globin vectors, essential viral components (packaging signal (0.4 kb), cPPT (0.1 kb), or RRE (0.8 kb)) were deleted in the backbone of the forward-oriented vector and the deleted viral components were inserted into the β-globin intron 2 (SspI site) between the splicing donor and branch site. To optimize a reverse-oriented globin vector, we deleted cryptic polyA signals by mutations in all AATAAA sequences, except for the LTR and β-globin downstream region, and deletions for the 5′-fragment of the promoter (482 bp) and the 3′-fragment of β-globin downstream region (331 bp)^42^. The standard marking vector contains an Mp promoter and GFP (or YFP) gene (pCL20cMpGFP), which was previously demonstrated to achieve efficient transduction in human hematopoietic repopulating cells in xenograft mice and rhesus hematopoietic repopulating cells in vivo^26–28^. The χHIV vectors were prepared by 293T cells (American Type Culture Collection (ATCC), Manassas, VA, USA) with co-transfection of chimeric gag-pol, rev-tat, VSVG envelope, and vector plasmids (6, 2, 2, and 10 μg, respectively), as previously described^26,28^. The media containing vectors were 100-fold concentrated by ultracentrifugation (82,700 × *g* (25k RPM in SW28 rotor) for 1.5 h, Optima XE-90, Beckman Coulter Life Sciences, Indianapolis, IN, USA). The GFP-encoding vector titers (IU/mL) were calculated by using GFP-positive percentages in transduced HeLa cells (when derived from Mp, ATCC) or MEL cells (when derived from the β-globin promoter, ATCC), evaluated by flow cytometry (FACSCalibur, BD Biosciences, East Rutherford, NJ, USA). The β-globin-encoding vector titers (no GFP marker) were calculated by VCNs in transduced HeLa cells in comparison with the GFP titer of a standard marking vector, evaluated by quantitative PCR (qPCR) (QuantStudio 6 Flex Real-Time PCR System, Thermo Fisher Scientific) with integration-specific self-inactivating-LTR probe/primers or LV2 probe/primers and TaqMan Ribosomal RNA control reagents (Thermo Fisher Scientific), as previously described^46^.

### Erythroid differentiation from transduced human CD34^+^ cells

Granulocyte colony-stimulating factor-mobilized CD34^+^ cells from healthy donors and plerixafor-mobilized CD34^+^ cells and steady-state PBMCs from SCD patients were collected under studies (08-H-0156, 17-H-0124, and 03-H-0015) that were approved by the Institutional Review Board of the National Heart, Lung, and Blood Institute (NHLBI). All individuals gave written informed consent for the sample donation and consent documents are maintained in the donor’s medical records. The consent document was approved by the Institutional Review Board prior to study initiation and is reviewed and updated yearly.

Human CD34^+^ cells were cultured in fibronectin (RetroNectin^TM^; Takara, Shiga, Japan)-coated 12-well plates with serum-free X-VIVO10 media (Lonza, Basel, Switzerland) containing 100 ng/ml each of stem cell factor (SCF, R&D Systems, Minneapolis, MN, USA), fms-related tyrosine kinase 3 ligand (R&D Systems), and thrombopoietin (R&D Systems)^26^. After overnight pre-stimulation, the cells were transduced with χHIV vectors at MOI 50 (or MOI de-escalation). The next day, transduced cells were differentiated into erythroid cells using Iscove’s modified Dulbecco’s medium (Mediatech, Inc., Manassas, VA)-based erythroid differentiation, including a 5- to 6-day differentiation phase with 20% fetal bovine serum (FBS, Mediatech), 2 U/ml erythropoietin (EPO, AMGEN, Thousand Oaks, CA, USA), 10 ng/ml SCF, 1.0 ng/ml interleukin 3 (R&D systems), 1.0 μM dexamethasone (VETone, Boise, ID, USA), and 1.0 μM estradiol (Pfizer, New York, NY, USA), and a subsequent 8- to 9-day maturation phase with 20% FBS, 2 U/ml EPO, 10 ng/ml insulin (Lilly, Indianapolis, IN, USA), 0.5 mg/ml transferrin (Sigma Aldrich, Saint Louis, MO, USA), and 2% bovine serum albumin (Roche, Indianapolis, IN, USA), which are slightly modified from human erythroid massive amplification culture^51,52^.

After erythroid differentiation, GFP-positive percentages in erythroid cells and GFP intensity in the GFP-positive fraction were evaluated by flow cytometry with glycophorin A (GPA) antibody (clone GA-R2, BD Biosciences). Hemoglobin production was evaluated by hemoglobin electrophoresis (Helena Laboratories, Beaumount, TX, USA)^52,53^.

### Xenograft transplantation of transduced human CD34^+^ cells

We used male NOD/SCID/IL2Rγ^null^ mice (NOD.Cg-Prkdc^scid^ IL2rg^tm1Wjl^/SzJ; Jackson Laboratory, Bar Harbor, ME, USA) that were 6–8 weeks old, following the guidelines set out by the Public Health Services Policy on Humane Care and Use of Laboratory Animals under a protocol approved by the Animal Care and Use Committee of the NHLBI. Human CD34^+^ cells (2 × 10^6^ cells per mouse) were pre-stimulated and transduced with lentiviral vectors at MOI 50, and these cells were injected into the NOD/SCID/IL2Rγ^null^ mice following sublethal busulfan conditioning (35 mg/kg, Busulfex, PDL BioPharma, Redwood City, CA, USA)^26^. The percentages of human CD45^+^ cells (clone HI30; BD Biosciences) and VCNs in human cells were evaluated in peripheral blood cells in the xenograft mice. Bone marrow cells were collected from the xenograft mice 4 months after transplantation, and these cells were cultured and differentiated into human erythroid cells using the same erythroid differentiation protocol from human CD34^+^ cells^52,54^. GFP expression among human GPA-positive erythroid cells was evaluated by flow cytometry.

### Rhesus HSC transplantation with lentiviral transduction

We previously developed a large animal model for HSC transplantation with lentiviral transduction in rhesus macaques, following the guidelines set out by the Public Health Services Policy on Humane Care and Use of Laboratory Animals under a protocol approved by the Animal Care and Use Committee of the NHLBI^27,28,55^. Granulocyte colony-stimulating factor (Amgen, Thousand Oaks, CA, USA) and SCF (Amgen)-mobilized rhesus CD34^+^ cells were transduced with χHIV vectors encoding GFP, YFP, or β-globin gene at MOI 50, and the transduced cells were transplanted into autologous rhesus macaques following total 10 Gy total body irradiation (2 × 5 Gy).

In competitive repopulation assays between GFP and YFP markers, GFP- or YFP-positive percentages in peripheral blood cells were evaluated by flow cytometry, whereas the GFP or YFP VCNs were evaluated by qPCR with GFP and YFP probe/primers^27,28^. Human β-globin production in rhesus RBCs was evaluated in flow cytometry with human adult hemoglobin antibody (clone H1)^56,57^. Human β-globin production was confirmed by RP-HPLC and LC-MS, as previously described^53^. Human β-globin RNA expression was evaluated by reverse-transcription qPCR using human β-globin-specific probe/primers (qhBG forward: 5′-GTG CCT TTA GTG ATG GCC TGG-3′, qhBG reverse: 5′-CCT GAA GTT CTC AGG ATC CAC G-3′, and qhBG probe: 5′-ACC TCA AGG GCA CCT TTG CCA CA-3′) and reference probe/primers detectable for both human and rhesus β-globins (qbothBG-f: 5′-CTC CTG ATG CTG TTA TGG G-3′, qbothBG-r: 5′-CTT GAG GTT GTC CAG GTG-3′, and qbothBG-p: 5′-ACT TTC TTG CCA TGA GCC TTC ACC TTA GGG-3′).

### Southern blot analysis

The integrated vector sizes were evaluated by Southern blot analysis, as previously described^58,59^. Briefly, DNA extracted from transduced HeLa cells were digested by NotI and EcoRI (New England Bio Labs, Beverly, MA, USA), and the sizes of digested DNA were determined by an HS2 probe (0.8 kb), which was produced by in vitro transcription from a PCR product of HS2F3 + T7p primer (5′-TAA TAC GAC TCA CTA TAG GGT GGG TGG ACT GCT TGG AGC TCA GGA GTT C-3′) and HS2R3 primer (5′-TAT GAT GCC GTT TGA GGT GGA G-3′). The band sizes are larger in intron-2-containing β-globin vectors (2.0–2.9 kb) than no intron-2 β-globin vectors (1.4 kb).

### Statistical analysis

Statistical analyses were performed using the JMP 13 software (SAS Institute, Inc., Cary, NC, USA). Two averages were evaluated with one-tailed Student’s *t*-test. The averages in various conditions were evaluated by Dunnett’s test (one-way analysis of variance (ANOVA) for a control) or Tukey’s honestly significant difference test (one-way ANOVA among multiple groups). A correlation was evaluated by *t*-test for coefficient of correlation and *R*^2^ in regression analysis. A *p*-value of <0.01 or 0.05 was deemed significant. SEM was shown as error bars in all figures.

## Supplementary information

Supplementary Information

## Data Availability

All relevant data are available from the corresponding author upon reasonable request. Human β-globin sequence is available at Genbank (NM_000518).

## References

[1] Charache S (1995). Effect of hydroxyurea on the frequency of painful crises in sickle cell anemia. Investigators of the Multicenter Study of Hydroxyurea in Sickle Cell Anemia. N. Engl. J. Med..

[2] Fitzhugh CD, Hsieh MM, Bolan CD, Saenz C, Tisdale JF (2009). Granulocyte colony-stimulating factor (G-CSF) administration in individuals with sickle cell disease: time for a moratorium?. Cytotherapy.

[3] Niihara Y (2014). A phase 3 study of l-glutamine therapy for sickle cell anemia and sickle ß0-thalassemia. Blood.

[4] Hsieh MM (2014). Nonmyeloablative HLA-matched sibling allogeneic hematopoietic stem cell transplantation for severe sickle cell phenotype. JAMA.

[5] Hsieh MM (2009). Allogeneic hematopoietic stem-cell transplantation for sickle cell disease. N. Engl. J. Med..

[6] Walters MC (1996). Bone marrow transplantation for sickle cell disease. N. Engl. J. Med..

[7] Allen ES (2017). Immunohaematological complications in patients with sickle cell disease after haemopoietic progenitor cell transplantation: a prospective, single-centre, observational study. Lancet Haematol..

[8] Aiuti A (2009). Gene therapy for immunodeficiency due to adenosine deaminase deficiency. N. Engl. J. Med..

[9] Cavazzana-Calvo M (2010). Transfusion independence and HMGA2 activation after gene therapy of human beta-thalassaemia. Nature.

[10] Hacein-Bey-Abina S (2003). A serious adverse event after successful gene therapy for X-linked severe combined immunodeficiency. N. Engl. J. Med..

[11] Ott MG (2006). Correction of X-linked chronic granulomatous disease by gene therapy, augmented by insertional activation of MDS1-EVI1, PRDM16 or SETBP1. Nat. Med..

[12] Cavazzana-Calvo M (2000). Gene therapy of human severe combined immunodeficiency (SCID)-X1 disease. Science.

[13] Boztug K (2010). Stem-cell gene therapy for the Wiskott-Aldrich syndrome. N. Engl. J. Med..

[14] May C (2000). Therapeutic haemoglobin synthesis in beta-thalassaemic mice expressing lentivirus-encoded human beta-globin. Nature.

[15] Miller AD, Bender MA, Harris EA, Kaleko M, Gelinas RE (1988). Design of retrovirus vectors for transfer and expression of the human beta-globin gene. J. Virol..

[16] Pestina TI (2009). Correction of murine sickle cell disease using gamma-globin lentiviral vectors to mediate high-level expression of fetal hemoglobin. Mol. Ther. J. Am. Soc. Gene Ther..

[17] Sadelain M, Wang CH, Antoniou M, Grosveld F, Mulligan RC (1995). Generation of a high-titer retroviral vector capable of expressing high levels of the human beta-globin gene. Proc. Natl Acad. Sci. USA.

[18] Malik P, Arumugam PI, Yee JK, Puthenveetil G (2005). Successful correction of the human Cooley’s anemia beta-thalassemia major phenotype using a lentiviral vector flanked by the chicken hypersensitive site 4 chromatin insulator. Ann. N. Y. Acad. Sci..

[19] Persons DA (2001). Functional requirements for phenotypic correction of murine beta-thalassemia: implications for human gene therapy. Blood.

[20] Puthenveetil G (2004). Successful correction of the human beta-thalassemia major phenotype using a lentiviral vector. Blood.

[21] Rivella S, May C, Chadburn A, Riviere I, Sadelain M (2003). A novel murine model of Cooley anemia and its rescue by lentiviral-mediated human beta-globin gene transfer. Blood.

[22] Imren S (2002). Permanent and panerythroid correction of murine beta thalassemia by multiple lentiviral integration in hematopoietic stem cells. Proc. Natl Acad. Sci. USA.

[23] Ribeil JA (2017). Gene therapy in a patient with sickle cell disease. N. Engl. J. Med..

[24] Kanter J (2017). Interim results from a phase 1/2 clinical study of lentiglobin gene therapy for severe sickle cell disease. Blood.

[25] Uchida N, Washington KN, Lap CJ, Hsieh MM, Tisdale JF (2011). Chicken HS4 insulators have minimal barrier function among progeny of human hematopoietic cells transduced with an HIV1-based lentiviral vector. Mol. Ther. J. Am. Soc. Gene Ther..

[26] Uchida N (2011). Optimal conditions for lentiviral transduction of engrafting human CD34+ cells. Gene Ther..

[27] Uchida N (2012). High-efficiency transduction of rhesus hematopoietic repopulating cells by a modified HIV1-based lentiviral vector. Mol. Ther. J. Am. Soc. Gene Ther..

[28] Uchida N (2009). Development of a human immunodeficiency virus type 1-based lentiviral vector that allows efficient transduction of both human and rhesus blood cells. J. Virol..

[29] Pawliuk R (2001). Correction of sickle cell disease in transgenic mouse models by gene therapy. Science.

[30] Evans ME (2014). TRIM5alpha variations influence transduction efficiency with lentiviral vectors in both human and rhesus CD34(+) cells in vitro and in vivo. Mol. Ther. J. Am. Soc. Gene Ther..

[31] Uchida N, Hsieh MM, Washington KN, Tisdale JF (2013). Efficient transduction of human hematopoietic repopulating cells with a chimeric HIV1-based vector including SIV capsid. Exp. Hematol..

[32] Fitzhugh CD (2017). At least 20% donor myeloid chimerism is necessary to reverse the sickle phenotype after allogeneic HSCT. Blood.

[33] Perumbeti A (2009). A novel human gamma-globin gene vector for genetic correction of sickle cell anemia in a humanized sickle mouse model: critical determinants for successful correction. Blood.

[34] Kwiatkowski JL (2017). Clinical outcomes up to 3 years following lentiglobin gene therapy for transfusion-dependent β-thalassemia in the Northstar Hgb-204 Study. Blood.

[35] Walters MC (2017). Results from the Hgb-207 (Northstar-2) Trial: a phase 3 study to evaluate safety and efficacy of lentiglobin gene therapy for transfusion-dependent β-thalassemia (TDT) in patients with non-β0/β0 genotypes. Blood.

[36] Leonard A (2017). Bone marrow characterization in sickle cell disease: inflammation and stress erythropoiesis lead to suboptimal CD34 recovery compared to normal volunteer bone marrow. Blood.

[37] Karn J, Stoltzfus CM (2012). Transcriptional and posttranscriptional regulation of HIV-1 gene expression. Cold Spring Harb. Perspect. Med..

[38] Uwimana N, Collin P, Jeronimo C, Haibe-Kains B, Robert F (2017). Bidirectional terminators in *Saccharomyces cerevisiae* prevent cryptic transcription from invading neighboring genes. Nucleic Acids Res..

[39] Maxwell IH, Brown JL, Maxwell F (1991). Inefficiency of expression of luciferase reporter from transfected murine leukaemia proviral DNA may be partially overcome by providing a strong polyadenylation signal. J. Gen. Virol..

[40] Le Guiner C (2014). Transgene regulation using the tetracycline-inducible TetR-KRAB system after AAV-mediated gene transfer in rodents and nonhuman primates. PLoS ONE.

[41] Elkon R, Ugalde AP, Agami R (2013). Alternative cleavage and polyadenylation: extent, regulation and function. Nat. Rev. Genet..

[42] Hanawa H (2004). Extended beta-globin locus control region elements promote consistent therapeutic expression of a gamma-globin lentiviral vector in murine beta-thalassemia. Blood.

[43] Schambach A, Galla M, Maetzig T, Loew R, Baum C (2007). Improving transcriptional termination of self-inactivating gamma-retroviral and lentiviral vectors. Mol. Ther. J. Am. Soc. Gene Ther..

[44] Boulad F (2014). Safe mobilization of CD34+ cells in adults with beta-thalassemia and validation of effective globin gene transfer for clinical investigation. Blood.

[45] Levasseur DN, Ryan TM, Pawlik KM, Townes TM (2003). Correction of a mouse model of sickle cell disease: lentiviral/antisickling beta-globin gene transduction of unmobilized, purified hematopoietic stem cells. Blood.

[46] Uchida N (2013). Integration-specific in vitro evaluation of lentivirally transduced rhesus CD34(+) cells correlates with in vivo vector copy number. Mol. Ther. Nucleic Acids.

[47] Uchida N (2019). High-efficiency lentiviral transduction of human CD34(+) cells in high-density culture with poloxamer and prostaglandin E2. Mol. Ther. Methods Clin. Dev..

[48] Uchida N (2011). Accelerated lymphocyte reconstitution and long-term recovery after transplantation of lentiviral-transduced rhesus CD34+ cells mobilized by G-CSF and plerixafor. Exp. Hematol..

[49] Hanawa H (2002). Comparison of various envelope proteins for their ability to pseudotype lentiviral vectors and transduce primitive hematopoietic cells from human blood. Mol. Ther. J. Am. Soc. Gene Ther..

[50] Hatziioannou T (2006). Generation of simian-tropic HIV-1 by restriction factor evasion. Science.

[51] Migliaccio G (2010). Humanized culture medium for clinical expansion of human erythroblasts. Cell Transplant..

[52] Uchida N (2018). Serum-free erythroid differentiation for efficient genetic modification and high-level adult hemoglobin production. Mol. Ther. Methods Clin. Dev..

[53] Uchida N (2017). Efficient generation of beta-globin-expressing erythroid cells using stromal cell-derived induced pluripotent stem cells from patients with sickle cell disease. Stem Cells (Dayt., Ohio).

[54] Hayakawa J (2010). The assessment of human erythroid output in NOD/SCID mice reconstituted with human hematopoietic stem cells. Cell Transplant..

[55] Donahue RE (1996). Peripheral blood CD34+ cells differ from bone marrow CD34+ cells in Thy-1 expression and cell cycle status in nonhuman primates mobilized or not mobilized with granulocyte colony-stimulating factor and/or stem cell factor. Blood.

[56] Hayakawa J (2009). Transient in vivo beta-globin production after lentiviral gene transfer to hematopoietic stem cells in the nonhuman primate. Hum. Gene Ther..

[57] Stanker, L. H., Branscomb, E., Vanderlaan, M. & Jensen, R. H. Monoclonal antibodies recognizing single amino acid substitutions in hemoglobin. *J. Immunol. (Baltimore, MD: 1950)* **136**, 4174–4180 (1986). 3701067

[58] Uchida N, Hanawa H, Yamamoto M, Shimada T (2013). The chicken hypersensitivity site 4 core insulator blocks promoter interference in lentiviral vectors. Hum. Gene Ther. Methods.

[59] Uchida N, Hanawa H, Dan K, Inokuchi K, Shimada T (2009). Leukemogenesis of b2a2-type p210 BCR/ABL in a bone marrow transplantation mouse model using a lentiviral vector. J. Nippon Med. Sch..

