# Supplementary material for: Development of a forward-oriented therapeutic lentiviral vector for hemoglobin disorders
Source: Nat Commun. 2019 Oct 2;10:4479. doi: 10.1038/s41467-019-12456-3 (PMC6775231; doi:10.1038/s41467-019-12456-3)
Supplement: Supplementary file 1 — Supplementary Information [file 41467_2019_12456_MOESM1_ESM.pdf]

Supplementary information

Supplementary figures

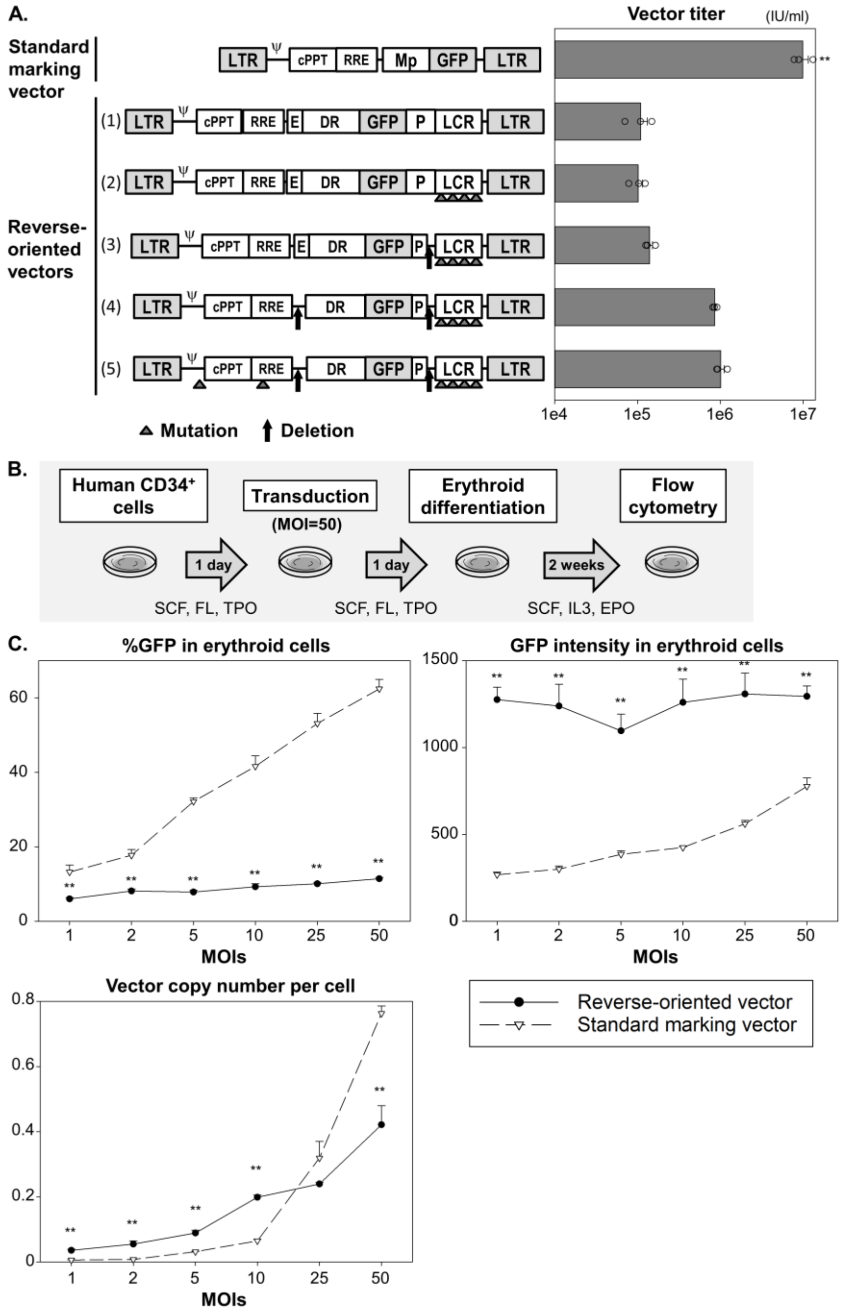

Supplementary figure 1. Lower vector titers and less efficient transduction in human $CD34^+$ cells with reverse-oriented vectors. (**A**) We deleted cryptic polyadenylation (polyA) signals to optimize a conventional reverse-oriented globin vector, and evaluated vector titers as compared to a standard marking vector encoding enhanced green fluorescent protein (GFP). **$p<0.01$ evaluated by Dunnett's test, compared to the optimized vector (5). (**B**) We transduced human $CD34^+$ cells with an optimized reverse-oriented vector (5) encoding GFP instead of globin gene at multiplicity of infection (MOI) 1-50, and the transduced cells were differentiated into erythroid cells. (**C**) Two weeks after erythroid differentiation, we evaluated transduction efficiency (GFP-positive percentages (%GFP)) in glycophorin A (GPA)-positive erythroid cells, GFP intensity in GFP-positive erythroid cells, and vector copy number per cell (VCNs). **$p<0.01$ evaluated by t-test. LTR: long terminal repeat, ψ: packaging signal, E: β-globin 3' enhancer, DR: β-globin downstream region, SCF: stem cell factor, FL: fms-related tyrosine kinase 3 ligand, TPO: thrombopoietin, IL3: interleukin 3, EPO: erythropoietin. All experiments were performed in triplicate.

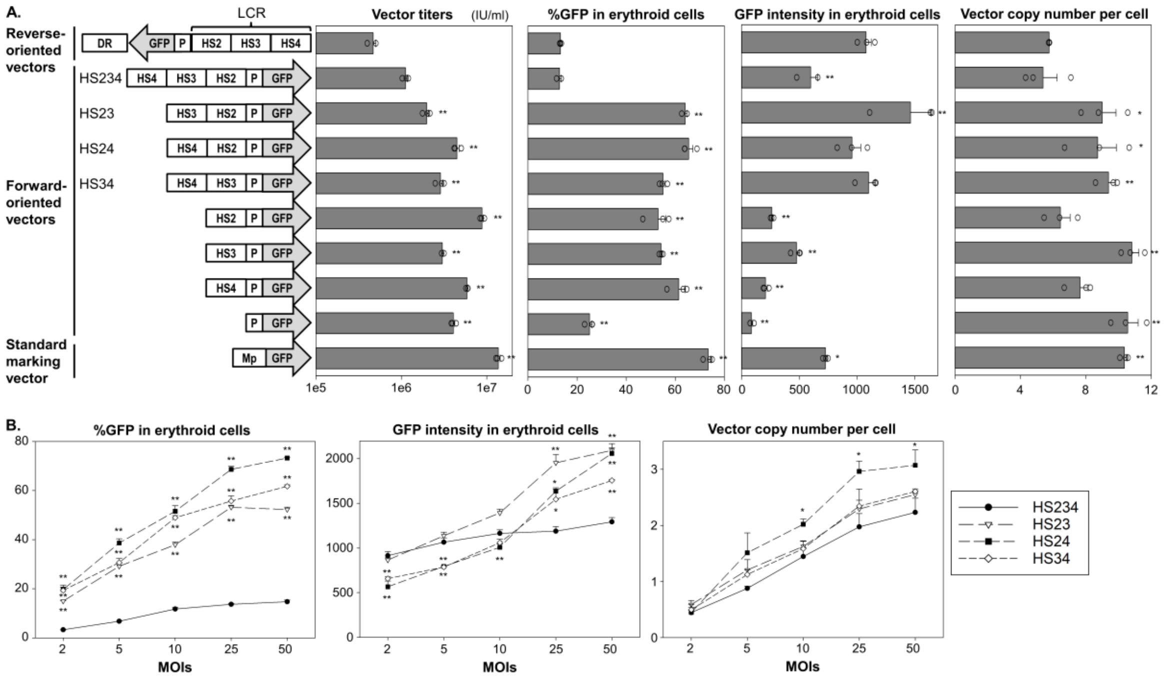

Supplementary figure 2. Higher vector titers and more efficient transduction in forward-oriented vectors with minimalizing LCR. (**A**) To further improve vector titers and transduction efficiency in human CD34⁺ cells, we designed various forward-oriented globin-expressing vectors, in which a globin expressing cassette (used in the optimized reverse-oriented vector) was inserted in the same orientation as the lentiviral vector backbone. We evaluated vector titers among all combinations of β-globin locus control region (LCR) composed of hypersensitive sites 2, 3, and 4 (HS2, HS3, and HS4) in a forward-oriented globin expression vector construct, as compared to the optimized reverse-oriented vector and a standard GFP marking vector. %GFP, GFP intensity, and VCNs were evaluated in human CD34⁺ cells transduced with the forward-

oriented vectors at MOI 50 followed by erythroid differentiation. **$p<0.01$, *$p<0.05$ evaluated by Dunnett's test, compared to the reverse-oriented vector. (**B**) We transduced human $CD34^+$ cells at MOI escalation of forward-oriented vectors with smaller sizes of LCR (HS23, HS24, and HS34) or full size LCR (HS234) followed by erythroid differentiation. **$p<0.01$, *$p<0.05$ evaluated by Dunnett's test, compared to the forward-oriented vector with HS234. DR: β-globin downstream region, P: β-globin promoter, Mp: murine stem cell virus promoter. All experiments were performed in triplicate.

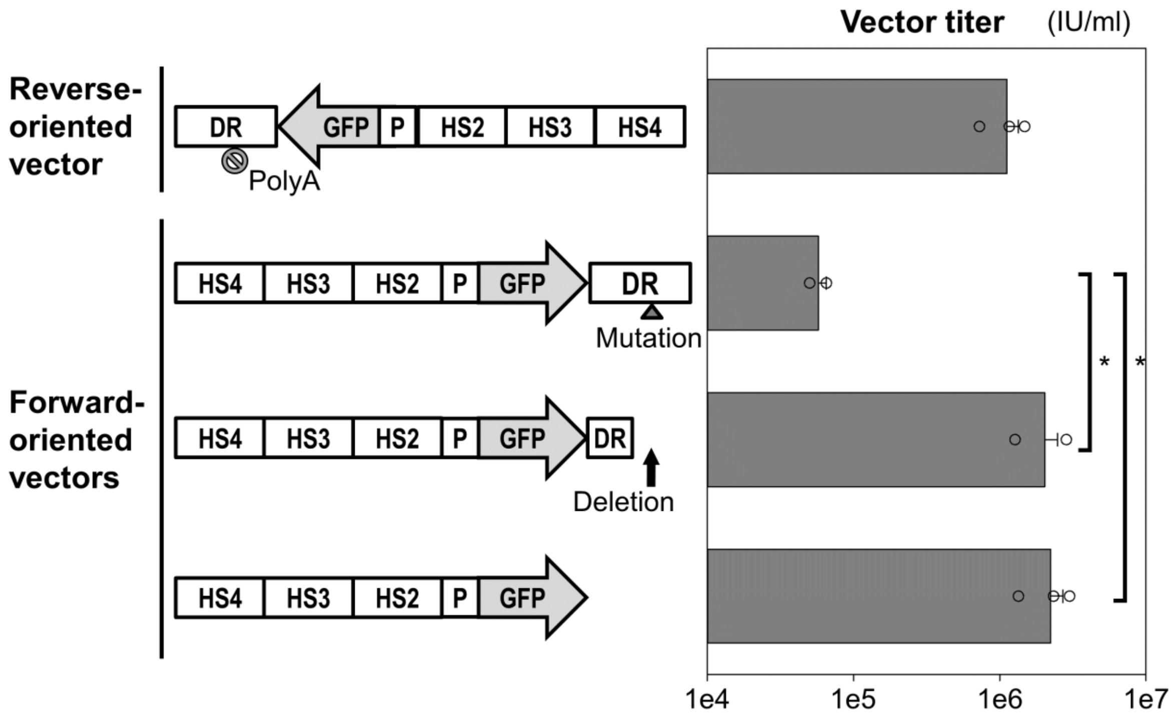

Supplementary figure 3. Lacking polyA function in the β-globin downstream region with a large deletion. To eliminate the polyA function in β-globin downstream region (with an enhancer function), we added an enhancer region of the β-globin downstream region with mutations of polyA signal or a large deletion of polyA signal region in a forward-oriented vector and evaluated vector titers. *$p<0.05$ evaluated by Tukey HSD test. All experiments were performed in triplicate.

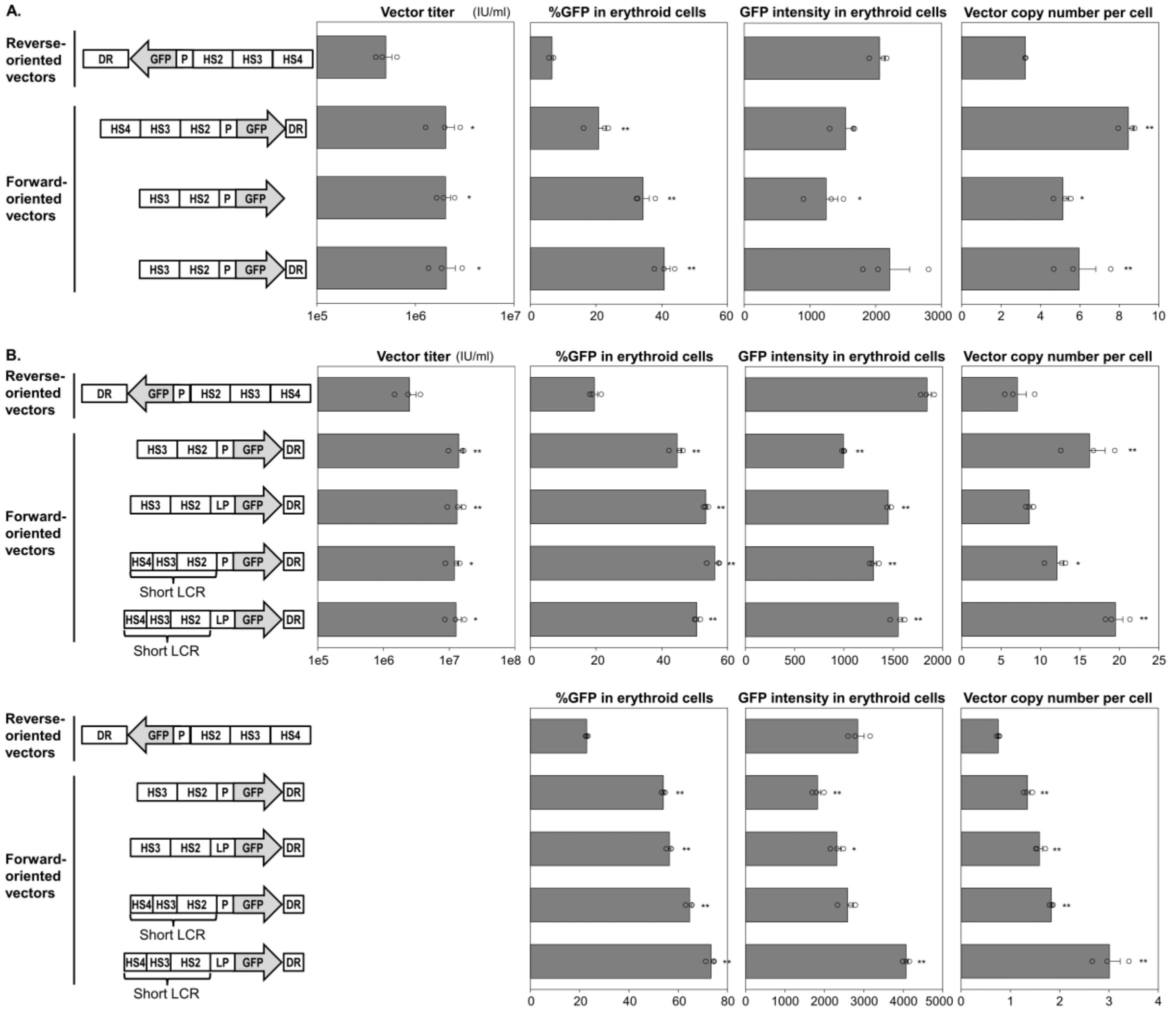

Supplementary figure 4. Higher transgene expression levels in forward-oriented vectors including a large segment of the β-globin promoter and an enhancer region of the β-globin downstream region lacking the polyA signal. (**A**) To increase transgene expression levels from forward-oriented vectors, we added the β-globin downstream region including the 3' untranslated region (3'UTR) and lacking the polyA signal (Supplementary figure 2). We transduced $CD34^{+}$ cells with these forward-oriented vectors, and the transduced cells were

differentiated into erythroid cells. **p<0.01, *p<0.05 evaluated by Dunnett's test, compared to the reverse-oriented vector. (**B**) Furthermore, we evaluated a longer β-globin promoter (LP) in forward-oriented vectors including HS23 or short LCR (HS2, small HS3, and small HS4) (repeated transduction results in bottom panel). **p<0.01, *p<0.05 evaluated by Dunnett's test, compared to the reverse-oriented vector. All experiments were performed in triplicate.

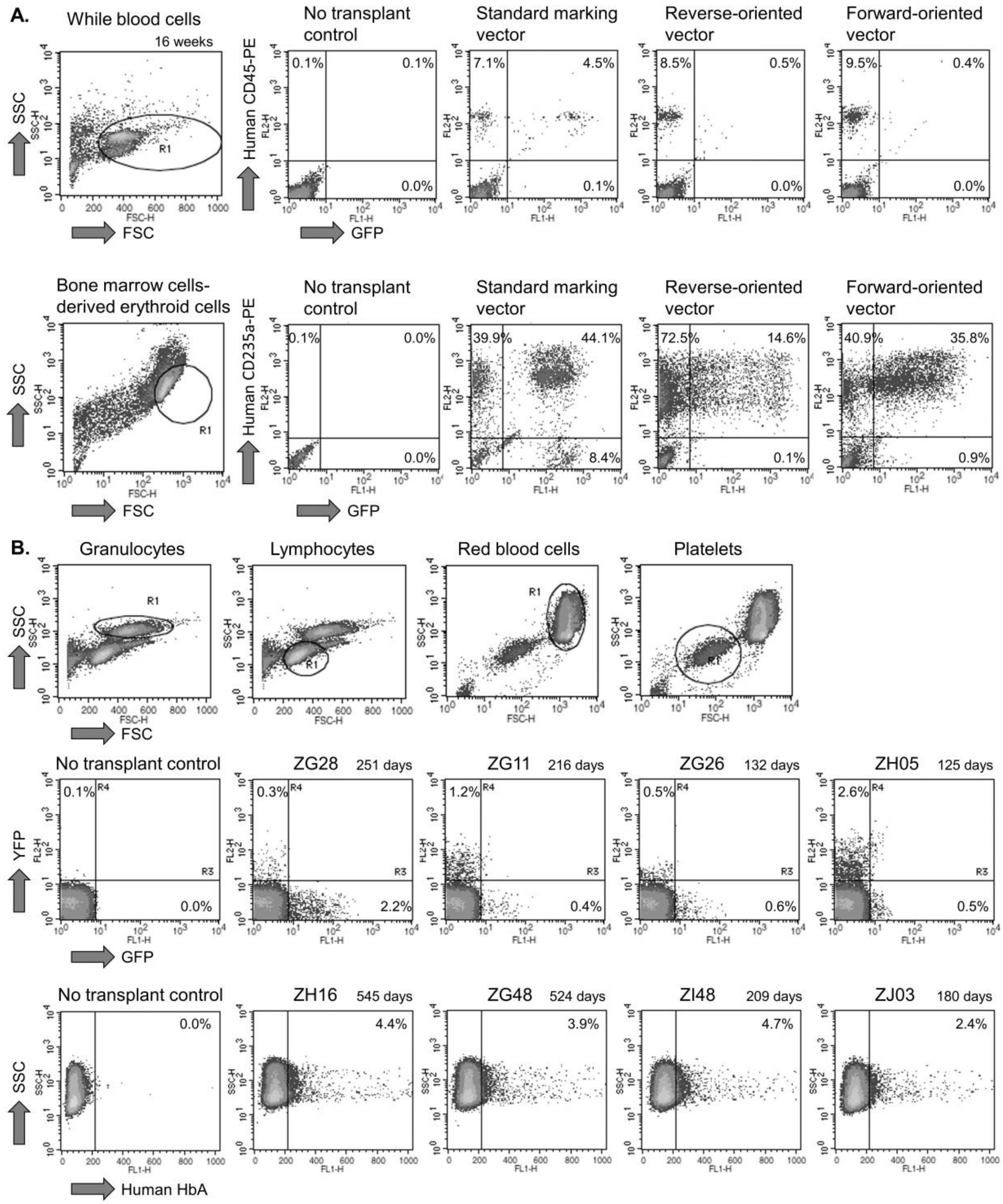

Supplementary figure 5. Flow cytometry panels to evaluate transgene expression from forward-oriented vectors in xenograft mouse and rhesus transplantation. (A) Human

CD45 and GFP expression was evaluated in a while blood cell fraction after xenograft transplantation (upper panels). After erythroid differentiation from xenograft bone marrow cells, human CD235a (glycophorin A) and GFP expression was evaluated (lower panels). (**B**) GFP and YFP expression (central panels) was separately evaluated in granulocyte, lymphocyte, red blood cell, and platelet fractions in transplanted rhesus macaques (upper panels), while human adult hemoglobin (HbA) expression was evaluated in the red blood cell fraction (lower panels). FSC: forward scatter, SSC: side scatter, PE: phycoerythrin.

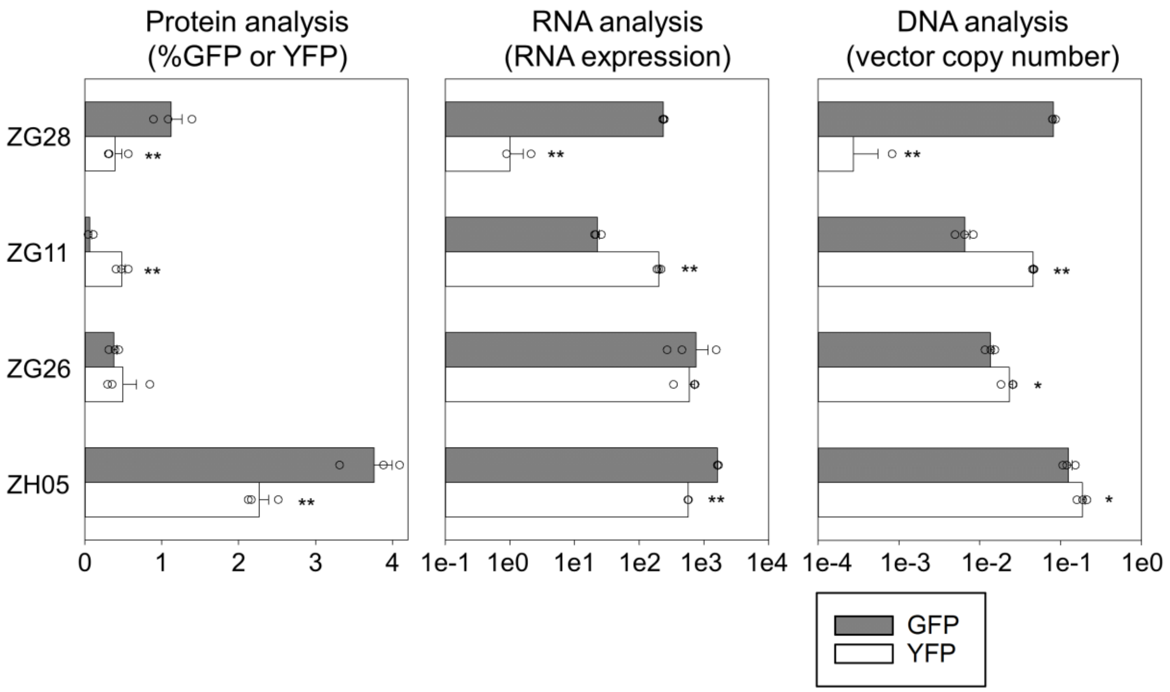

Supplementary figure 6. Comparison of GFP and YFP by protein, RNA, and DNA analysis among erythroid cells differentiated from transplanted macaques. We differentiated peripheral blood mononuclear cells from transplanted macaques (ZG28, ZG11, ZG26, and ZH05) into erythroid cells, and evaluated GFP and YFP signals by protein (by flow cytometry), RNA (by reverse transcription-quantitative PCR), and DNA (by quantitative PCR) levels. **p<0.01, *p<0.05 evaluated by t-test. All experiments were performed in triplicate.

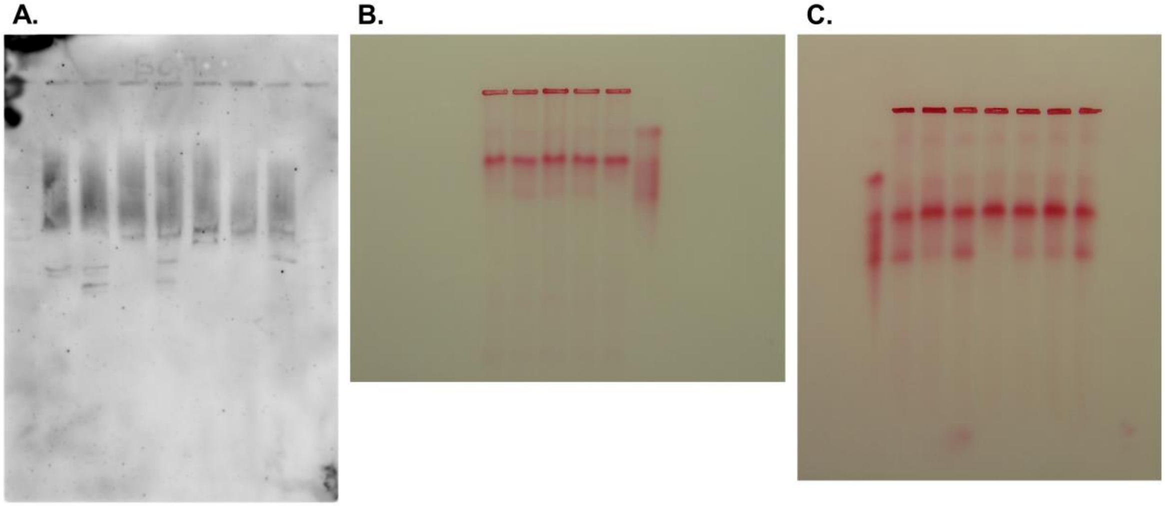

Supplementary figure 7. Original images of Southern blot analysis and hemoglobin electrophoresis. (A) An original image of Southern blot analysis to evaluate integrating vector sizes, used in Figure 4B. **(B)** An original image of hemoglobin electrophoresis to confirm β-globin expression from the HS234 forward-oriented vector. The 2nd, 3rd, 5th, and 6th lanes were used in Figure 4C. **(C)** An original image of hemoglobin electrophoresis to evaluate β-globin expression from the HS1-5 forward-oriented vector. The 1st, 5th, 6th, 7th, and 8th lanes were used in Figure 6A.

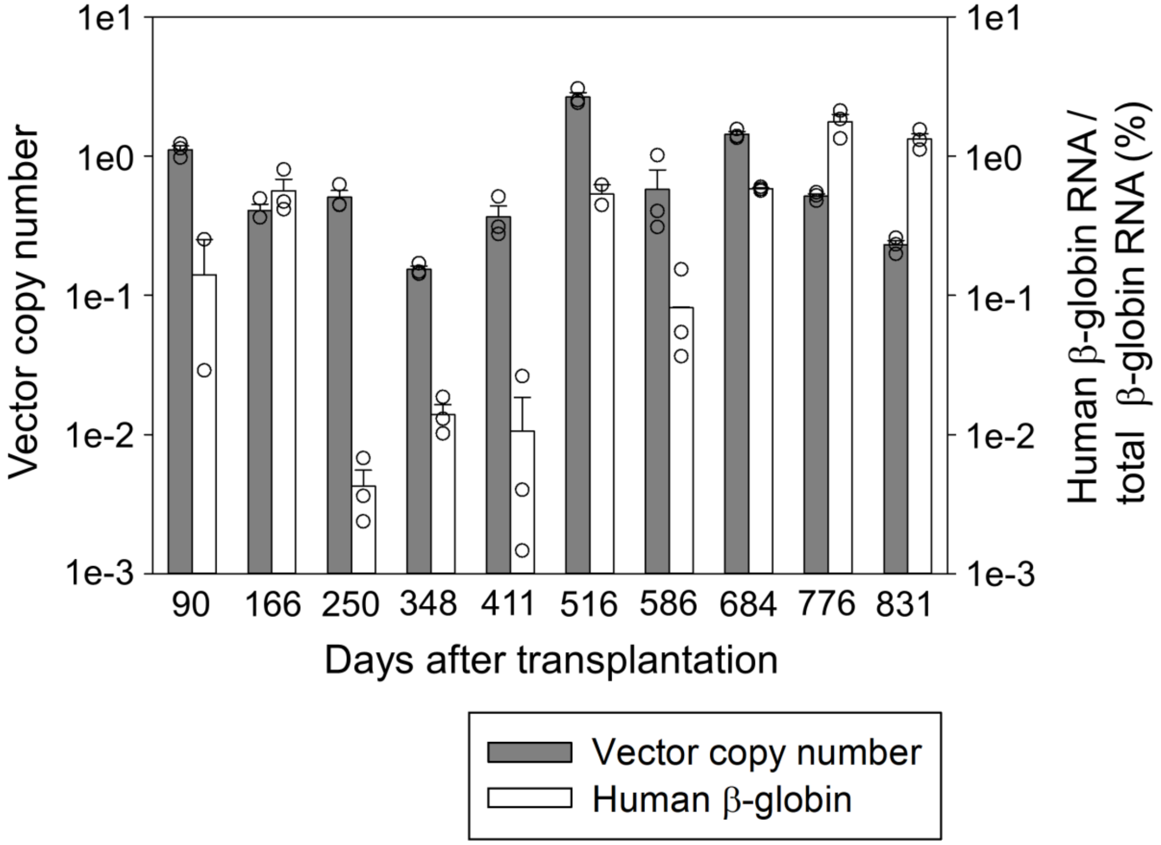

Supplementary figure 8. Detectable human β-globin expression at the RNA level with the forward-oriented β-globin vector in rhesus transplantation. The human globin RNA expression (by reverse transcription-quantitative PCR) and VCNs (by quantitative PCR) were evaluated in bone marrow mononuclear cells after transplantation of rhesus $CD34^+$ cells (ZI48) transduced with the forward-oriented globin vector. All experiments were performed in triplicate.

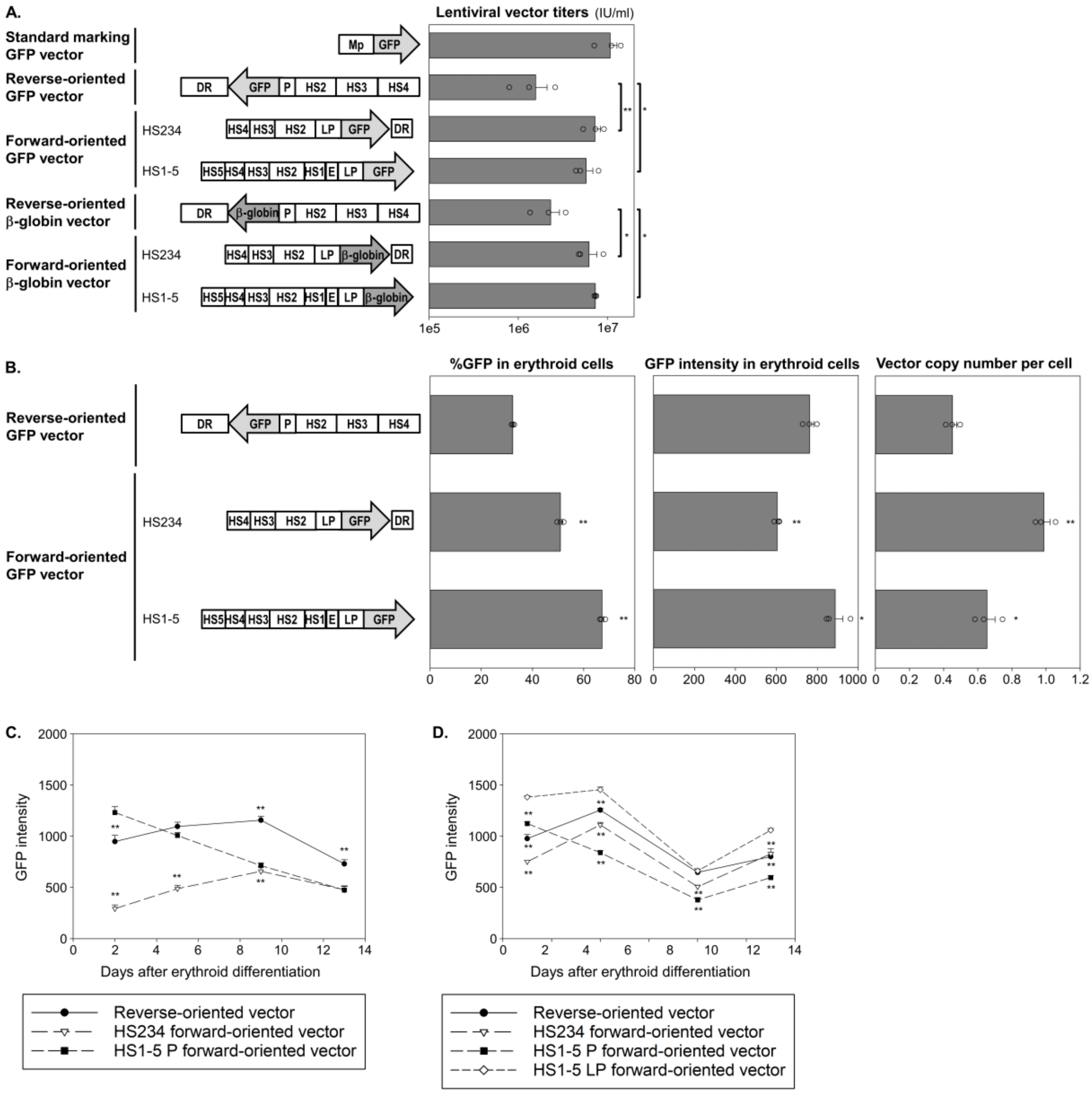

Supplementary figure 9. Robust transgene expression from a forward-oriented vector with additional hypersensitive sites 1 and 5 (HS1 and HS5) and β-globin 3' enhancer (E).

(A) To further improve transgene expression levels from forward-oriented vectors, we added HS1, HS5, and E (with 3'UTR deletion), and evaluated vector titers in GFP-encoding and β-globin-

encoding vectors without ultracentrifugation. **p<0.01, *p<0.05 evaluated by Dunnett's test, compared to the reverse-oriented vector. **(B)** %GFP, GFP intensity, and VCNs were evaluated in transduced human CD34⁺ cells followed by erythroid differentiation. **p<0.01, *p<0.05 evaluated by Dunnett's test, compared to the reverse-oriented vector. **(C and D)** GFP intensity was evaluated during erythroid differentiation from transduced human CD34⁺ cells among the reverse-oriented vector, HS234 forward-oriented vector, HS1-5 P forward-oriented vector (including the small-size β-globin promoter (P)), and HS1-5 LP forward-oriented vector (including the large-size β-globin promoter (LP)). **p<0.01, *p<0.05 evaluated by Dunnett's test, compared to the HS1-5 P forward-oriented vector (C) or the HS1-5 LP forward-oriented vector (D). All experiments were performed in triplicate.

Supplementary table

Supplementary table 1. *In vitro* transduction efficiency in rhesus CD34⁺ cells which were transplanted into autologous animals.

Animal ID	Lentiviral vectors	Vector copy number	%GFP or %YFP

		per cell	in erythroid cells
ZG28	Forward-oriented GFP vector	2.3	64.0
	Reverse-oriented YFP vector	10.9	42.9
ZG11	Forward-oriented YFP vector	7.1	63.4
	Reverse-oriented GFP vector	2.2	41.2
ZG26	Forward-oriented GFP vector	2.2	74.7
	Standard marking YFP vector	3.4	63.6
ZH05	Forward-oriented YFP vector	4.1	79.6
	Standard marking GFP vector	0.8	52.5
ZH16	Forward-oriented β-globin vector	3.4	N.A.
ZG48	Forward-oriented β-globin vector	8.6	N.A.
ZI48	Forward-oriented β-globin vector	6.7	N.A.

ZJ03	Forward-oriented β-globin vector	1.3	N.A.

N.A.: not applicable.

Supplementary note

Supplementary note 1. Lower vector titers and less efficient transduction in human CD34⁺ cells with reverse-oriented vectors for globin expression

Current globin expressing vectors are all reverse-oriented to prevent loss of intron 2 by RNA splicing during viral preparation, since intron 2 is required for high-level β-globin expression [1]. We hypothesized that reverse-orientation impedes both viral preparation and vector transduction. We optimized a conventional reverse-oriented globin vector encoding enhanced green fluorescent protein (GFP) instead of the globin gene by deleting cryptic polyadenylation (polyA) signals [2], and evaluated vector titers in transduced MEL cells (Supplementary figure 1A). Optimization resulted in 10-fold increase of vector titers, mainly due to a 0.3kb deletion of the β-globin 3' enhancer; however, titers were still 10-fold lower than a standard GFP marking vector which was previously demonstrated to achieve high-level gene marking for human and rhesus

hematopoietic repopulating cells [3, 4, 5]. We then transduced human CD34$^+$ cells with an optimized reverse-oriented vector (including 6 mutations and 3 deletions to eliminate potential cryptic polyA signals) at multiplicity of infection (MOI) 1-50, and 2 weeks after erythroid differentiation, evaluated transduction efficiency (GFP-positive percentages) in glycophorin A (GPA)-positive erythroid cells (Supplementary figure 1B). We observed more efficient transduction at increasing MOIs with a standard marking vector, while transduction efficiency with the reverse-oriented vector was similar for all MOIs, resulting in much lower transduction efficiency for the reverse-oriented vector at MOI 50 as compared to the standard marking vector ($p<0.01$) (Supplementary figure 1C). Similarly, VCNs in the standard marking vector increased more sharply at higher MOIs as compared to the reverse-oriented vector (Supplementary figure 1C). GFP intensities in the reverse-oriented vector were higher than the standard marking vector at all MOIs, since the reverse-oriented vector contains a strong enhancer and promoter to express the transgene specifically in erythroid cells. However, the GFP intensities in the standard making vector increased at higher MOIs, while similar GFP intensities were observed among MOIs in the reverse-oriented vector (Supplementary figure 1C). These data suggest that even after optimization, the required reverse-orientation of the globin-expression cassette impedes both viral preparation and vector transduction, and further improvement is needed for efficient

transduction with globin vectors in human HSCs.

Supplementary note 2. Higher vector titers and more efficient transduction with forward-oriented vectors for globin expression

For further improvement of both vector titers and transduction efficiency in $CD34^+$ cells, we designed forward-oriented globin-expressing vectors, in which a globin-expression cassette (used in the optimized reverse-oriented vector) was inserted in the same orientation as the HIV-1 vector backbone (Supplementary figure 2A). All components in the globin-expression cassette were optimized for the forward-orientation, including the LCR, β-globin promoter, and β-globin downstream region. The LCR enhances transcription from the β-globin promoter (P), and it is composed of hypersensitive site (HS) 2, HS3, and HS4 in reverse-oriented globin vectors. To begin, we evaluated the following combinations of the LCR (3.2kb) in the forward-orientation: a full size LCR (HS234), double HS (HS23, HS24, and HS34), single HS (HS2, HS3, and HS4), and no HS. All forward-orientated vectors demonstrated higher vector titers as compared to the optimized reverse-oriented vector ($p<0.01$ except HS234). After erythroid differentiation of transduced $CD34^+$ cells (MOI 50), more efficient transduction was observed among forward-

oriented vectors including smaller sized LCR components ($p<0.01$ double HS, single HS, and no HS), while minimal sized LCRs (single HS and no HS) resulted in lower transgene expression ($p<0.01$), as compared to the reverse-oriented vector. In summary, longer LCRs increased GFP intensities in forward-oriented vectors, but GFP intensities were reduced by insertion of HS234 due to less efficient transduction. At MOI escalation for human $CD34^+$ cell transduction with the forward-oriented vectors (HS234 and double HS), more efficient transduction was observed at increasing MOIs, while smaller sized LCR vectors (double HS) resulted in higher transduction efficiency as compared to full sized LCR vectors (HS234) ($p<0.01$ at all MOIs) (Supplementary figure 2B). Among these smaller sized LCR vectors (double HS), higher transgene expression was observed from the HS23 combination than the HS24 and HS34 combinations and was similar to a full size LCR vector (HS234) at low MOIs (MOIs 2-5).

In addition, we observed 1.2-2.0 fold higher VCNs but 1.3-7.0 fold lower GFP intensities in the no HS vector and single HS vectors (HS2, HS3, and HS4) as compared to the HS234 vector (Supplementary figure 2A), demonstrating lower transgene expression from the no HS and single HS forward-oriented vectors. In contrast, the double HS vector (HS23, HS24, and HS34) transduction (MOI 50) resulted in 1.6-1.7 fold higher VCNs and 1.6-2.4 fold higher GFP intensities, demonstrating higher transduction efficiency in the double HS forward-oriented

vectors. In low MOI setting (MOI 2 at MOI escalation) with similar VCNs among double HS and HS234 vectors, similar GFP intensity in the HS23 vector and lower GFP intensity in the HS24 and HS34 vectors were observed as compared to the HS234 vector (Supplementary figure 2B), demonstrating similar transgene expression from the HS23 forward-oriented vector and lower transgene expression from the HS24 and HS34 vectors.

We then evaluated the β-globin downstream region (0.7kb) including 3'UTR and polyA signal, since the lower transgene expression observed was likely due to absence of the downstream region in the forward-oriented vector with a full sized LCR (HS234) as compared to the optimized reverse-oriented vector. We deleted the 3' site of the β-globin downstream region to eliminate the polyA function, since the polyA signal reduced vector titers in the forward-orientation even with mutations in the AATAAA site ($p<0.05$) (Supplementary figure 3) [6]. The addition of a small version of the β-globin downstream region (0.1kb) including the 3'UTR and lacking the polyA signal didn't change either vector titer (Supplementary figures 3 and 4A) or transduction efficiency (%GFP in erythroid cells and VCNs) in $CD34^+$ cells (Supplementary figure 4A, between HS23 vectors with and without the 0.1kb β-globin downstream region), while higher transgene expression was observed in the forward-oriented vector including the small β-globin

downstream region (with a smaller sized LCR HS23), which was similar to the reverse-oriented vector (with a full-sized LCR and β-globin downstream region) (Supplementary figure 4A). Though lower overall vector titers were obtained in this cycle of vector preparation based on variability of culture conditions, we again observed significantly higher vector titers in all forward-oriented vectors than those with the optimized reverse-oriented vector ($p<0.05$). In addition, we evaluated a larger size of the β-globin promoter (LP) (0.7kb) in the forward-oriented vectors including the HS23 combination (2.1kb) as well as a short LCR (2.1kb) composed of HS2, small HS3, and small HS4 (a smaller size of triple HS combination) (Supplementary figure 4B). Inclusion of the large-size promoter (LP) and/or the short LCR of the triple HS combination (short HS234) resulted in higher transgene expression (1.2-1.5 fold and 1.1-1.3 fold, respectively) in the forward-oriented vector with high vector titers ($p<0.01$) and efficient transduction (%GFP in erythroid cells ($p<0.01$) and VCNs ($p<0.05$ except the HS23 vector including LP)) (Supplementary figure 4B). Although transgene expression levels were slightly lower in the forward-oriented vectors as compared to the reverse-oriented vector in this condition ($p<0.01$), the LP and short HS234 combination resulted in the highest transgene expression among the forward-oriented vectors.

Based on these data, the optimized forward-oriented globin vector was comprised of a short LCR of triple HS combination (short HS234), a large size β-globin promoter (LP), and the β-globin downstream region (including 3'UTR) lacking the polyA signal and was prepared with 100-fold concentration for further animal experiments (Figure 1A). The concentrated vector titers of the forward-oriented vectors ($1.0 \pm 0.2 \times 10^9$ IU/mL) were still 6-fold higher than the optimized vector in the reverse-orientation ($1.6 \pm 0.2 \times 10^8$ IU/mL, $p<0.01$), and comparable to a standard GFP-marking vector ($1.9 \pm 0.2 \times 10^9$ IU/mL). These data demonstrate that the optimized forward-oriented vectors for globin expression has higher vector titers and more efficient transduction in $CD34^+$ cells for erythroid differentiation than the optimized reverse-oriented vector.

Supplementary note 3. High-level transgene expression from a forward-oriented vector with additional hypersensitive sites 1 and 5 (HS1 and HS5) and β-globin 3' enhancer.

To further improve transgene expression levels from the formerly optimized forward-oriented vector (HS234), we added HS5 (inserted in upstream of HS4), HS1 (inserted in downstream of HS2), and 0.3kb β-globin 3' enhancer (inserted in upstream of β-globin promoter), reduced the sizes of HS3 (0.7kb HS3) and HS4 (0.2kb HS4), and deleted β-globin 3'UTR to

generate the HS1-5 forward-oriented vector (Supplementary figure 9). The total transgene expression cassette size in the HS1-5 forward-oriented vector is similar (0.2kb larger) to the HS234 forward-oriented vector. Both HS1-5 and HS234 forward-oriented vectors have higher vector titers as compared to the reverse-oriented vector ($p<0.05$), while similar viral titers were observed in both HS1-5 and HS234 forward-oriented vectors (Supplementary figure 9A). We transduced human $CD34^+$ cells at MOI 50 followed by erythroid differentiation, resulting in higher transduction efficiency (%GFP, $p<0.01$ and VCNs, $p<0.05$) in both HS234 and HS1-5 forward-oriented vectors, compared to the reverse-oriented vector (Supplementary figure 9B). The HS234 forward-oriented vector has lower transgene expression levels (GFP intensity, $p<0.01$) as compared to the reverse-oriented vector, while slightly higher gene expression ($p<0.05$) was observed in the HS1-5 forward-oriented vector, compared to the reverse-oriented vector (Supplementary figure 9B). These data suggest that inclusion of greater numbers of HS fragments improves gene expression from the forward-oriented vector.

In addition, we evaluated GFP expression levels during erythroid differentiation from transduced human $CD34^+$ cells. When the HS234 forward-oriented vector including the large-size β-globin promoter (LP) was compared with the HS1-5 forward-oriented vector including the

small-size β-globin promoter (P) which was used in the reverse-oriented β-globin vector, higher GFP intensity was observed in the HS1-5 P vector than the HS234 vector during the early phase of erythroid differentiation ($p<0.01$); however, GFP intensity in the HS1-5 P vector was reduced during the late phase of erythroid differentiation (Supplementary figure 9C). We then inserted LP into the HS1-5 forward-oriented vector instead of P. In the HS1-5 LP forward-oriented vector, GFP intensity was increased not only during the early phase but also the late phase of erythroid differentiation (Supplementary figure 9D), resulting in higher GFP intensity for the entire duration of erythroid differentiation ($p<0.01$) as compared to the HS234 forward-oriented vector. These data demonstrate that inclusion of LP can enhance gene expression from the forward-oriented vector during both the early and late phases of erythroid differentiation. The HS1-5 LP forward-oriented vector allows for high vector titer, high-efficiency transduction in human $CD34^+$ cells, and high-level transgene expression in erythroid cells.

Supplementary discussion

The road to gene therapy for the globin disorders has been long and winding, and β-globin vectors have been in development for more than 30 years. The initial vectors were

designed to include a β-globin cDNA using γ-retroviral vectors in the forward-orientation; however, β-globin production was undetectable with these preliminary vectors. In 1988, Miller and colleagues convincingly demonstrated the necessity of inclusion of intron 2 of β-globin for achieving detectable β-globin expression which has dictated a reverse-orientation for β-globin vectors since [1]. Inclusion of a large sized LCR was reported to further increase γ-globin expression in a reverse-oriented vector [7]. These elements proved difficult to include in γ-retroviral vectors , and lentiviral vectors were eventually chosen over these γ-retroviral vectors to improve the stability of vector genomes including a reverse-oriented β-globin expressing cassette [8]. Once the reverse-oriented β-globin vector was established in a lentiviral vector system, research turned to evaluating the vector's therapeutic effects in mouse models of β-thalassemia and SCD [8, 9, 10, 11, 12, 13, 14], and the necessity of reverse-orientation for high-level β-globin expression was confirmed. Beginning now 10 years ago, we started to optimize a β-globin vector for efficient transduction in our rhesus gene therapy model, since we reached therapeutic gene marking levels in the rhesus model with a standard marking lentiviral vectors (including a small, constitutive promoter and GFP cDNA). However, our optimized reverse-oriented vector could not achieve a sufficient level of transduction of human or rhesus $CD34^+$ cells when compared to this standard marking lentiviral vector. Therefore, we hypothesized that the reverse-orientation

required for β-globin vectors interferes with efficient transduction of $CD34^+$ cells, and we thus developed a forward-oriented β-globin vector with an RRE-based intron 2 selection for high-efficiency transduction and high-level β-globin expression.

To facilitate our work to develop these optimized β-globin vectors with high-efficiency transduction and high-level transgene expression in erythroid cells, we developed an efficient and reliable screening system by first incorporating a fluorescent reporter (GFP) that allowed us to analyze GFP expression (instead of β-globin) in erythroid cells differentiated from transduced human $CD34^+$ cells. This circumvented the need for the more cumbersome globin expression analysis for the screening that would require quantitative polymerase chain reaction (qPCR), as well as hemoglobin electrophoresis and reverse phase HPLC. We could thus move more swiftly, introducing a therapeutic gene into only the best constructs. In addition, as VCN data are PCR generated, smaller differences are more difficult to reliably demonstrate, whereas flow cytometry allows the more sensitive analysis required for these important optimization steps. Additionally, GFP analysis allowed us to obtain not only objective flow cytometry data (GFP-positive percentages and GFP intensity), but also subjective fluorescent microscopy images that allow us to confirm these data by simple microscopy. The combination of objective and subjective

analyses can increase the confidence in our gene expression data. After these optimization steps to develop the forward-oriented vector, we confirmed high-level VCNs in animal models (Figures 1, 2, 3, and 5) and robust β-globin expression in erythroid cells differentiated from SCD $CD34^+$ cells (Figure 6).

A number of new technologies have opened the window to several additional and elegant genetic strategies that could potentially cure SCD. Fetal hemoglobin, which has anti-sickling effects, can now be reliably reactivated via lentiviral gene therapy (gene addition) with an RNA interference for BCL11A knockdown as well as a looping molecule for binding between β-globin locus control region (enhancer) and γ-globin promoter [15, 16]. Genome editing technologies can also induce fetal hemoglobin expression by targeting genes associated with hereditary persistence of fetal hemoglobin, including the erythroid specific enhancer for BCL11A and the BCL11A binding site upstream of γ-globin promoter as just two examples [17, 18]. In addition, conversion from the SCD mutation to the wild-type β-globin sequence (gene correction) can now be achieved using genome editing technologies [18]. These genetic strategies are being investigated in our preclinical evaluation models, including *in vitro* erythroid differentiation, humanized xenograft mouse transplantation, and the rhesus HSC gene therapy model.

In preliminary clinical trials for γ-retroviral gene therapy, several patients with immunodeficiencies developed leukemia due to insertional mutagenesis [19, 20, 21]. To reduce insertional mutagenesis and improve safety, gene delivery vectors were switched from γ-retroviral vectors to lentiviral vectors [7, 22], and a self-inactivating long terminal repeat (SIN-LTR) was used to remove promoter and enhancer elements from the intact LTR [23]. In current gene therapy trials for β-thalassemia and SCD, no leukemia development has been reported with reverse-oriented β-globin vectors including a SIN-LTR. In the initial trial for β-thalassemia, a dominant clone with a vector integration into HMGA2 gene was detected; however, after a long-term follow-up, the percentage of the dominant clone decreased and other dominant clones were not clonally expanded [24]. In all other gene therapy trials for immunodeficiency diseases, forward-oriented vectors were used, and no genotoxicity was reported with SIN lentiviral vectors. Our forward-oriented β-globin vector contains a SIN-LTR, and no leukemia development was observed for 4 years after transplantation of transduced $CD34^+$ cells, suggesting that the forward-oriented β-globin vector has a favorable safety profile comparable to lentiviral vectors currently in clinical trials.

Supplementary references

1. Miller AD, Bender MA, Harris EA, Kaleko M, Gelinas RE. Design of retrovirus vectors for transfer and expression of the human beta-globin gene. *Journal of virology* **62**, 4337-4345 (1988).

2. Hanawa H, Hargrove PW, Kepes S, Srivastava DK, Nienhuis AW, Persons DA. Extended beta-globin locus control region elements promote consistent therapeutic expression of a gamma-globin lentiviral vector in murine beta-thalassemia. *Blood* **104**, 2281-2290 (2004).

3. Uchida N*, et al.* High-efficiency transduction of rhesus hematopoietic repopulating cells by a modified HIV1-based lentiviral vector. *Molecular therapy : the journal of the American Society of Gene Therapy* **20**, 1882-1892 (2012).

4. Uchida N, Hsieh MM, Hayakawa J, Madison C, Washington KN, Tisdale JF. Optimal conditions for lentiviral transduction of engrafting human CD34+ cells. *Gene therapy* **18**, 1078-1086 (2011).

5. Uchida N*, et al.* Development of a human immunodeficiency virus type 1-based lentiviral vector that allows efficient transduction of both human and rhesus blood cells. *Journal of virology* **83**, 9854-9862 (2009).

6. Jiang Y, Xu XS, Russell JE. A nucleolin-binding 3' untranslated region element stabilizes beta-globin mRNA in vivo. *Molecular and cellular biology* **26**, 2419-2429 (2006).

7. Mitchell RS*, et al.* Retroviral DNA integration: ASLV, HIV, and MLV show distinct target site preferences. *PLoS biology* **2**, E234 (2004).

8. May C*, et al.* Therapeutic haemoglobin synthesis in beta-thalassaemic mice expressing lentivirus-encoded human beta-globin. *Nature* **406**, 82-86 (2000).

9. Imren S*, et al.* Permanent and panerythroid correction of murine beta thalassemia by multiple lentiviral integration in hematopoietic stem cells. *Proc Natl Acad Sci U S A* **99**, 14380-14385 (2002).

10. Malik P, Arumugam PI, Yee JK, Puthenveetil G. Successful correction of the human Cooley's anemia beta-thalassemia major phenotype using a lentiviral vector flanked by the chicken hypersensitive site 4 chromatin insulator. *Ann N Y Acad Sci* **1054**, 238-249 (2005).

11. Persons DA, Allay ER, Sabatino DE, Kelly P, Bodine DM, Nienhuis AW. Functional requirements for phenotypic correction of murine beta-thalassemia: implications for human gene therapy. *Blood* **97**, 3275-3282 (2001).

12. Pestina TI, Hargrove PW, Jay D, Gray JT, Boyd KM, Persons DA. Correction of murine sickle cell disease using gamma-globin lentiviral vectors to mediate high-level expression of fetal hemoglobin. *Molecular therapy : the journal of the American Society of Gene Therapy* **17**, 245-252 (2009).

13. Puthenveetil G*, et al.* Successful correction of the human beta-thalassemia major phenotype using a lentiviral vector. *Blood* **104**, 3445-3453 (2004).

14. Rivella S, May C, Chadburn A, Riviere I, Sadelain M. A novel murine model of Cooley anemia and its rescue by lentiviral-mediated human beta-globin gene transfer. *Blood* **101**, 2932-2939 (2003).

15. Sankaran VG*, et al.* Developmental and species-divergent globin switching are driven by BCL11A. *Nature* **460**, 1093-1097 (2009).

16. Breda L*, et al.* Forced chromatin looping raises fetal hemoglobin in adult sickle cells to higher levels than pharmacologic inducers. *Blood* **128**, 1139-1143 (2016).

17. Bauer DE*, et al.* An erythroid enhancer of BCL11A subject to genetic variation determines fetal hemoglobin level. *Science* **342**, 253-257 (2013).

18. Traxler EA*, et al.* A genome-editing strategy to treat beta-hemoglobinopathies that recapitulates a mutation associated with a benign genetic condition. *Nat Med* **22**, 987-990 (2016).

19. Hacein-Bey-Abina S*, et al.* Insertional oncogenesis in 4 patients after retrovirus-mediated gene therapy of SCID-X1. *J Clin Invest* **118**, 3132-3142 (2008).

20. Ott MG*, et al.* Correction of X-linked chronic granulomatous disease by gene therapy, augmented by insertional activation of MDS1-EVI1, PRDM16 or SETBP1. *Nat Med* **12**, 401-409 (2006).

21. Aiuti A*, et al.* Gene therapy for immunodeficiency due to adenosine deaminase deficiency. *N Engl J Med* **360**, 447-458 (2009).

22. Wu X, Li Y, Crise B, Burgess SM. Transcription start regions in the human genome are favored targets for MLV integration. *Science* **300**, 1749-1751 (2003).

23. Ryu BY, Evans-Galea MV, Gray JT, Bodine DM, Persons DA, Nienhuis AW. An experimental system for the evaluation of retroviral vector design to diminish the risk for proto-oncogene activation. *Blood* **111**, 1866-1875 (2008).

24. Cavazzana-Calvo M*, et al.* Transfusion independence and HMGA2 activation after gene therapy of human beta-thalassaemia. *Nature* **467**, 318-322 (2010).
